# Stimbiotic supplementation and xylose-rich carbohydrates modulate broiler’s capacity to ferment fibre

**DOI:** 10.3389/fmicb.2023.1301727

**Published:** 2024-01-11

**Authors:** Claire Davies, Gemma González-Ortiz, Teemu Rinttilä, Juha Apajalahti, Mohammad Alyassin, Michael R. Bedford

**Affiliations:** ^1^AB Vista, Wiltshire, United Kingdom; ^2^Alimetrics Research Ltd., Espoo, Finland; ^3^School of Applied Sciences, University of Huddersfield, Huddersfield, United Kingdom

**Keywords:** fibre fermentation, xylooligosaccharide, degree of polymerisation, ileal microbiota, caecal microbiota, SCFA, *ex vivo*

## Abstract

Stimbiotics are a new category of feed additives that can increase fibre fermentability by stimulating fibre-degrading microbiota in the gut. The aim of this study was to test, *ex vivo*, if the microbiota of broilers fed a stimbiotic are better able to ferment different xylose-rich substrates in an ileal and a caecal environment. The ileal and caecal contents from broiler chickens fed a stimbiotic or from a control group were used as an inoculum in the *ex vivo* fermentation experiment. Different xylose-rich substrates including monomeric xylose (XYL), XOS with DP 2 to 6 (XOS), short DP XOS of 2 to 3 (sDP-XOS), long DP XOS of 4 to 6 (lDP-XOS) and de-starched wheat bran (WB), were added to each ileal and caecal inoculum in fermentation vessels. Total gas, short-chain fatty acids (SCFA) production, bacterial quantification, and carbohydrate utilisation were monitored for 9 h post-inoculation. No significant interactions were observed in any of the parameters measured in either the ileal or caecal contents (*p* > 0.05). Stimbiotic ileal inocula resulted in higher total gas (*p* < 0.001) and volatile fatty acid (VFA) (*p* < 0.001) production, increased numbers of *Lactobacillus* spp. (*p* < 0.001), and decreased numbers of *Enterococcus* spp. (*p* < 0.01) after 9 h regardless of the xylose-rich substrate added. Stimbiotic caecal inocula resulted in a higher ratio of VFA to branched-chain fatty acids (BCFAs) by up to +9% (*p* < 0.05). Ileal microbiota were found to preferentially metabolise WB, while caecal microbiota favoured XOS substrates, particularly lDP-XOS. These results indicate that stimbiotics can promote the abundance of lactic acid bacteria involved in the establishment of fibre-degrading bacteria and VFA content in the gut, which could have beneficial effects on broiler performance. Further, ileal and caecal microbiota differ in their utilisation of different substrates which may impact the effectiveness of different stimbiotic products.

## 1 Introduction

The poultry gut microbiome is a complex and dynamic community of microorganisms that plays a major role in the health and productivity of poultry. The gut microbiome is responsible for a number of important functions including fibre fermentation, production of essential nutrients and energy, inhibition of pathogen colonisation, and development of the host’s immune system (Carrasco et al., 2019). In broiler chickens, microbiota can ferment fibre in the distal ileum and the caecum. The microbial communities in these two intestinal compartments are distinct. The ileal microbial community is simple, with lactobacilli making up 80–90% of the bacteria. The primary metabolite of lactobacilli, lactic acid, plays a major role in lowering the pH of the ileum and preventing colonisation by pH-sensitive pathogenic bacteria (Rinttilä and Apajalahti, 2013). Dietary nutrients and microbial metabolites that are not absorbed in the small intestine can reach the lower intestine and caecum where they serve as a substrate for a much denser and more diverse microbial population. The primary end products of microbial fermentation are short chain fatty acids (SCFAs), including lactic acid and volatile fatty acids (VFAs) such as acetate, propionate and butyrate. In addition to serving as an energy source for the host, SCFAs are involved in maintaining intestinal function.

One way to promote a functional gut microbiome is through dietary supplementation with stimbiotics. Stimbiotics are a relatively new class of non-digestible feed additives that promote dietary fibre utilisation by the commensal microbiota through a smooth transition process (González-Ortiz et al., 2019). In contrast to prebiotics, stimbiotics are added at such low doses, effective as low as 50 g/t (Morgan et al., 2023), that they contribute little to the production of SCFAs through direct fermentation but instead are thought to signal fibre-digesting bacterial species to become more active and increase the fermentation of fibre already present in the diet (Ribeiro et al., 2018). Studies have shown that xylo-oligosaccharides (XOS) act as stimbiotics by selectively stimulating the growth and activity of beneficial bacteria commonly found in the gut such as *Lactobacillus* spp. and *Bifidobacterium* spp. (Okazaki et al., 1990; Courtin et al., 2008; Wang et al., 2010). XOS has been linked to improved fibre digestion, higher SCFA concentration, and better gut health in poultry (Morgan et al., 2019, 2022; Šimić et al., 2023) and swine (Liu et al., 2018; González-Solé et al., 2022). However, the typical assumption is that microbial adaptation to XOS occurs mainly in the caecum while the ileum remains largely understudied.

Much remains unknown about the mode of action of stimbiotics. *In vivo* studies are often complicated by individual variation between birds and the challenge of measuring transient intermediate metabolites, which are rapidly taken up by the host. *Ex vivo* studies offer a more controlled process where live microbial communities can be transplanted from the gut into a culture vessel. This approach allows conditions to be regulated and matched to the target intestinal segment, whilst fermentation dynamics can be monitored in real-time without interference from epithelial absorptive processes (Bedford and Apajalahti, 2018; Apajalahti and Rinttilä, 2019). In the present study, an *ex vivo* system was used to investigate whether feeding a stimbiotic to broiler chickens can modulate the microbiome and improve its fibre fermentation capabilities when exposed to different xylose-rich carbohydrate substrates. In addition, the extent to which different XOS and xylose-based products were fermented by gut bacteria from the ileum and caecum was investigated. The study aimed to test the following hypotheses: (1) microbiota from stimbiotic-supplemented birds have a higher preference for XOS fermentation over monomeric xylose or wheat bran; (2) microbiota from stimbiotic-supplemented birds have higher activity overall; (3) microbiota from the ileum are more specialised to metabolise simple carbohydrates (e.g., xylose, XOS, short degree polymerisation XOS) while microbiota from the caecum are more capable of metabolising more complex carbohydrates (long degree polymerisation XOS and wheat bran).

## 2 Materials and methods

### 2.1 Xylose-rich carbohydrate substrates

**Figure 1:**
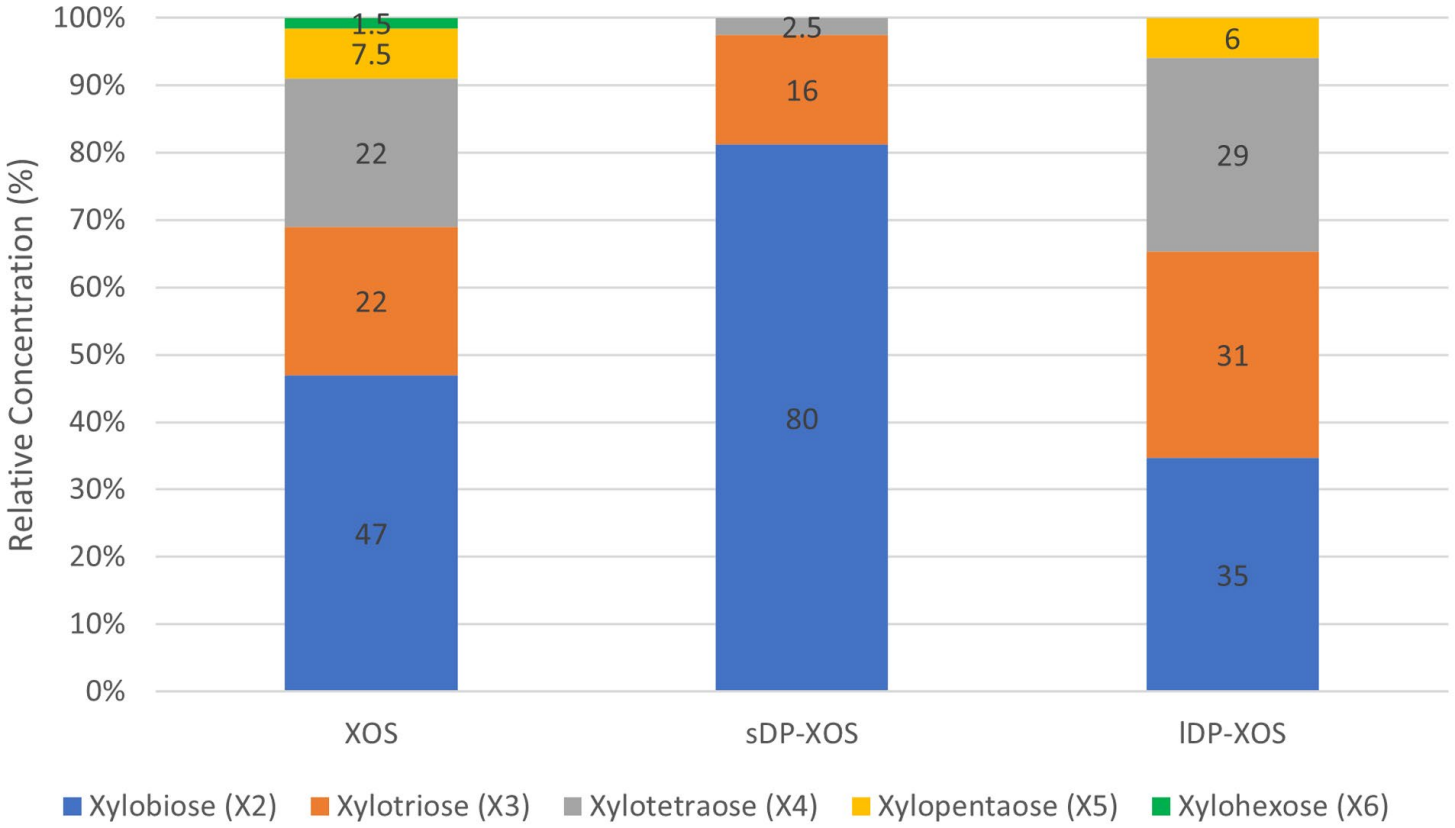
Relative concentration (%) of different length xylooligosaccharides in XOS substrates used in the study.

The xylose-rich carbohydrate substrates used in this study include: (1) monomeric D-xylose purchased from Sigma-Aldrich (St. Louis, MO, United States) with a purity of ≥99% (XYL); (2) xylo-oligosaccharides with degree of polymerisation (DP) between 2 and 6 (XOS); (3) xylo-oligosaccharides with a higher proportion of short DP (sDP-XOS); (4) xylo-oligosaccharides with a higher proportion of long DP (lDP-XOS); and (5) de-starched wheat bran (WB). The DP compositions of the different XOS substrates are shown in Figure 1. The sugar compositions of all substrates are shown in Table 1.

**Table 1:** Xylose-rich carbohydrate substrates used in the study.

	Xylose	XOS	sDP-XOS	lDP-XOS	Wheat bran
Xylose	5.00	2.55	3.56	5.05	0.20
Arabinose	0.00	0.29	0.54	0.31	0.11
Mannose	0.00	0.22	0.23	0.23	0.20
Galactose	0.00	0.29	0.28	0.40	0.19
Glucose	0.00	0.86	0.57	1.68	0.35

Sugar composition determined after acid hydrolyisis (mg/mL).

XOS, sDP-XOS, and lDP-XOS were provided by the School of Applied of Science, Huddersfield University (UK). Twenty grams of a corncob-derived XOS at 35% of purity and a degree of polymerisation (DP) between 2 and 6, was placed in a conventional Soxhlet extraction thimble using absolute ethanol (Fisher, Loughborough, UK) as a solvent. The extraction was carried out for 72 h with 32 extraction cycles per hour. The extraction solution was collected and dried, then redissolved in water and labelled as sDP-XOS. The remaining XOS in the substrate was then extracted with an 80% ethanol solution. The extraction solution was centrifuged at 4000 rpm for 30 min, filtered, evaporated to remove all the ethanol and excess water, concentrated, and labelled as lDP-XOS. The original corncob-derived XOS product was purified with 80% ethanol, recovered following the same steps of lDP-XOS, and labelled as XOS. All three extracts, sDP-XOS, lDP-XOS, and XOS, were analysed using the HPAEC-PAD method described in previous work (Alyassin et al., 2020). The analysis showed that sDP-XOS contained 80% xylobiose (X2), 16% xylotriose (X3) and 2.5% xylotetraose (X4), lDP-XOS contained 35% X2, 31% X3, 29% X4 and 6% xylopentaose (X5), and XOS contained 47% X2, 22% X3, 22% X4, 7.5% X5 and 1.5% xylohexaose (X6) (Figure 1).

De-starched WB was prepared enzymatically from commercial untreated WB (Korpelan Mylly Oy, Finland). Briefly, WB was suspended in 1:10 (w/v) sodium phosphate – bicarbonate buffer (pH 7.0) and the suspension was vigorously shaken for 15 min. Then 50 μL of heat-stable α-amylase (Sigma-Aldrich, St. Louis, MO, USA) was added and the suspension was incubated at 60°C for 60 min und er constant stirring, after which the sample was equilibrated at 37°C. The second step of de-starching was performed by adding 50 μL of amyloglucosidase (Sigma-Aldrich, St. Louis, MO, USA) to the suspension and incubated at 37°C for 30 min under constant stirring. After cooling to room temperature, the de-starched wheat sample was centrifuged (10,000 × g, 20 min, +4°C) and washed twice with deionised water prior to freeze-drying of the WB pellet.

### 2.2 *Ex vivo* incubation model

Ileum and caecum *ex vivo* fermentation experiments were conducted to mimic the luminal conditions of the ileum and caecum of broiler chickens. To prepare an authentic substrate medium for ileum fermentation, distal jejunal contents from 30 four-week-old Ross 308 broiler chickens fed a wheat-soya based diet without special additives (control birds) were collected, pooled, and homogenised. For the caecum fermentation, the substrate was prepared by combining distal ileal and caecal contents of the same birds. The jejunal and ileal/caecal digesta preparations were combined with an equal volume of pH 6.85 anaerobic buffer solution (0.02 M K_2_HPO_4_, 0.02 M NH_4_H_2_PO_4_, 0.6 mmol MgSO_4_) and centrifuged at 18,000 × g for 20 min to pellet the solids and bulk of bacteria. The pellet was discarded, and the clarified supernatants were used as the bacterial growth substrates in the *ex vivo* experiment.

The microbial inocula introduced for the respective ileum and caecum fermentation models comprised of fresh ileal or caecal digesta from four-week-old Ross 308 broilers fed either the wheat-soy-based control diet (Supplementary Table S1) or control diet amended with 100 g/t of stimbiotic product (Signis, AB Vista). No antibiotics, coccidiostats or other feed additives were included in these diets. Six control diet birds and six stimbiotic-fed birds were sacrificed. The intestinal contents were collected and maintained under anoxic conditions to retain viability of the bacteria until they were used for the inoculation of the simulation vessels (within 2 h of sacrificing the birds). The ileum simulation was initiated by adding 5.0 mL of the ileal substrate medium previously described, 50 mg of the xylose-rich carbohydrates, 0.1 g of ileal digesta inoculum of individual birds, and 5.0 mL of pH 6.85 buffer solution (20 mM K_2_HPO_4_, 20 mM NH_4_H_2_PO_4_, 0.6 mM MgSO_4_) to 20-ml serum bottles in an anaerobic chamber. The caecum simulation was initiated by adding 5.0 mL of the caecal substrate medium as previously described, 50 mg of the xylose-rich carbohydrates, 0.5 g of caecal digesta inoculum of individual birds and 5.0 mL of pH 6.85 buffer solution (20 mM K_2_HPO_4_, 20 mM NH_4_H_2_PO_4_, 0.6 mM MgSO_4_) to 20-ml serum bottles in an anaerobic chamber. To replicate genuine caecum conditions, the buffer was reduced to −340 mV with a mixture of NaS and cysteine.

Simulation vessels were sealed with thick butyl rubber stoppers, heated to 42°C, and continuously mixed in a gyratory shaker at 100 rpm. Each treatment was replicated in six vessels and the blank control was replicated in twelve vessels. These parallel vessels were considered biological replicates as the inocula were not pooled prior to introduction to simulation vessels. Instead, they were derived from individual birds fed either with the control or stimbiotic diet. The inoculation was done in a random order to avoid any potential systematic shifts or bias resulting from time of inoculation or freshness of the inoculum. The incubation of both the ileum and caecum simulations lasted for 9 h before the vessels were sampled for various analyses, as described below.

### 2.3 Sample analysis

#### 2.3.1 Total gas production

Total gas production was measured in the simulation vessels at 3-h intervals by manually puncturing the rubber stopper with a needle that was connected to a high-precision glass syringe with a sensitive ground plunger and recording the volume of total gas released from the fermentation vessels.

#### 2.3.2 Fermentation end-products

Volatile fatty acids (VFA) and lactic acid were analysed as free acids in both the ileum and caecum simulation vessels after 9-h incubation using pivalic acid (Sigma-Aldrich, St. Louis, MO, United States) as an internal standard (Apajalahti et al., 2019). For this, 400 μL of sample and 2.4 mL of 1.0 mM pivalic acid solution were mixed, shaken vigorously for 5 min, and then centrifuged at 3000 × g for 10 min. Then, 800 μL of the supernatant was mixed with 400 μL of saturated oxalic acid solution, incubated at 4°C for 60 min, and then centrifuged at 18,000 × g for 10 min. The supernatant was analysed by gas chromatography (Agilent Technologies, Santa Clara, CA, USA) using a glass column packed with 80/120 Carbopack B-DA/4% Carbowax stationary phase, helium as the carrier gas, and a flame ionization detector. The quantified acids were acetic, propionic, butyric, valeric, isobutyric, 2-methylbutyric, isovaleric, and lactic acid.

#### 2.3.3 Extraction of microbial DNA from ileum simulation samples

Subsamples from the ileum *ex vivo* experiment were collected for analysis of the abundance of different microbial species or genera using real-time quantitative PCR (qPCR). Microbial DNA was extracted from ileum simulation samples using the following protocol. First, 0.4 mL of each simulation sample was suspended in 1.6 mL of 50 mM phosphate-buffered saline with EDTA. Then, the suspension was shaken vigorously for 2 min. Next, the microcentrifuge tubes were centrifuged at 18,000 × g for 10 min to pellet the microbial cells. The pellet was resuspended in 600 μL of phosphate lysis buffer containing 100 mM Tris and 50 mM EDTA (pH 8.0). The suspension was transferred to a screw-cap microcentrifuge tube containing 20 μL of proteinase K (20 mg/mL; Roche Diagnostics GmbH, Mannheim, Germany) and 0.4 g of sterile glass beads (Scientific Industries Inc., Bohemia, NY, United States), and incubated at 65°C for 60 min with shaking for 30 s (1,400 rpm) at 10 min intervals. The microbial cells were disrupted by two 1 min rounds of bead beating (FastPrep-24™, MP Biomedicals, Irvine, CA, United States) at 6.5 m/s. Genomic DNA was then purified from the homogenates through phenol–chloroform–isoamyl alcohol (24:1) extraction with centrifugation at 10,000 × g for 10 min, followed by chloroform–isoamyl alcohol purification with centrifugation at 10,000 × g for 10 min. The DNA was precipitated by adding 0.6 volumes of 100% isopropanol and pelleted by centrifugation at 18,000 × g for 10 min. Finally, the DNA pellet was washed twice with 1 mL of ice-cold 70% ethanol, dried, and resuspended in 100 μL of Tris-EDTA buffer containing 10 mM Tris and 1.0 mM EDTA (pH 8.0) (AppliChem, Darmstadt, Germany).

#### 2.3.4 qPCR analysis of ileal bacteria

The qPCR assays used in the study were selected based on prior knowledge of the consistently dominant bacteria found in the small intestines of broilers at Alimetrics Research experimental broiler facility. qPCR analyses were performed using an ABI Prism Sequence Detection System 7,500 instrument (Thermo Fisher Scientific Inc., Waltham, MA, United States). Amplifications were conducted in 15 μL volumes with SYBR Select Master Mix (Thermo Fisher Scientific Inc., Waltham, MA, USA), 0.25 μM of both primers, and 5 μL of 1:100 diluted template DNA or deionized sterile water as a no-template control. The rRNA gene-targeted primer sequences and PCR product sizes used for enumeration of the target microorganisms are listed in Table 2. Amplification, standard, and melt curves for unpublished primers are provided in Supplementary Figure S1. The qPCR assay development and optimization were conducted as described in Rinttilä et al. (2020). The thermal cycling conditions involved one cycle of preheating at 50°C for 2 min and an initial denaturation at 95°C for 10 min, followed by 40 cycles of denaturation at 95°C for 15 s and annealing and extension at the primer-specific annealing temperatures for 60s. To verify the specificity of amplifications based on the melting temperature of PCR products, a melt curve analysis was carried out alongside each qPCR run. This involved slowly decreasing the temperature from 95 to 60°C, with fluorescence determination at 0.3°C intervals.

**Table 2:** Real-time quantitative PCR assays used in the study.

Target microorganism or group	Primer sequence (5′-3′)	Product size (bp)	Reference
Total eubacteria	F: TCCTACGGGAGGCAGCAGT	466	Nadkarni et al. (2002)
	R: GGACTACCAGGGTATCTAATCCTGTT		
*Lactobacillus* spp.	F: AGCAGTAGGGAATCTTCCA	341	Rinttila et al. (2004)
	R: CACCGCTACACATGGAG		
*Streptococcus* spp.	F: GGGGATAACTATTGGAAACGATA	118	Rinttilä et al. (2022)
	R: CCWACTAGCTAATACAACGCA		
*Enterococcus* spp.	F: AYCAACCTGCCCTTCAGA	147	Unpublished
	R: GCRACTCGTTGTACTTCC		
*Escherichia coli*	F: GGAGTAAAGTTAATACCTTTGCTC	214	Unpublished
	R: CCTCTACGAGACTCAAGCTT		
*Lactobacillus salivarius*	F: TTTACTCTCTGTTAAAGAATGGCTTA	146	Unpublished
	R: GAGCTAAGGCCCCATAAGAA		
*Lactobacillus crispatus*	F: CATGCAAGTCGAGCGAGC	448	Unpublished
	R: AATAAAGGCCAGTTACTACCTCTATC		
*Lactobacillus reuteri*	F: TGGCCCAACTRATTGATGG	188	Unpublished
	R: CATCCCAGAGTGATAGCCA		

Amplification, standard, and melt curves for unpublished primers are provided in Supplementary Figure S1.

In each 96-well plate, synthetic small-subunit rRNA gene copies of the target microorganism (gBlocks® Gene Fragments, IDT, Coralville, IA, United States) were included in tenfold serial dilutions ranging from 1 × 10^8^ to 1 × 10^2^. The fractional cycle number at which the fluorescence passed the 0.3 fluorescence unit threshold was determined for the unknowns and compared with the standard curves. By accounting for the original volume of the starting material, elution volume, and PCR template dilution, the numbers of small subunit rRNA gene targets were determined per ml of ileum simulation sample. For data analysis, the rRNA gene copies/mL values were log_10_-transformed to achieve a normal distribution.

#### 2.3.5 Carbohydrate analysis of ileal fermentation vessels and tested XOS products

For sugar analysis in the ileal simulation vessels after 9 h of incubation as well as xylose-rich test products, 0.5 g of sample was diluted in 4 mL of ice-cold water containing erythritol as an internal standard and vortexed for 5 min to extract soluble carbohydrates. Insoluble material was then spun down by centrifugation at 3,000 × g for 5 min. Two 200-ml aliquots were taken from the supernatant, one for the analysis of soluble monosaccharides (simple sugars) and the other for the analysis of total soluble carbohydrates.

For the determination of total soluble carbohydrates, the sample underwent acid hydrolysis by adding 200 mL of 2 moL/L H_2_SO_4_, sealing the vessel, and incubating at 100°C for 2 h. Subsequently, the hydrolysate was neutralised by adding 800 mL of 1 moL/L NaOH solution. The analysis of monosaccharides was performed on the original 200-ml sample without hydrolysis, but it was diluted with 1,000 mL of the H_2_SO_4_-NaOH solution to achieve the same ionic strength as the hydrolysed sample. 400 μL of the sample preparations were mixed with 800 mL of methanol to precipitate inorganic impurities, then 200 mL of the supernatant was evaporated to dryness. The monosaccharides in samples were converted to the corresponding oximes and derivatised with N,O-bis(trimethylsilyl)trifluoroacetamide as described by Zhang et al. (2018). Finally, the samples were analysed by gas chromatography mass spectroscopy (GC–MS) (Agilent 7,890-5975C GC-MSD equipped with a ZB-5 60 m × 250 mm × 0.25 mm column). Data was collected with a single ion mode and procedural calibration standards were used for quantification.

#### 2.3.6 Statistical analysis

The statistical analyses were performed using JMP 16 Pro (SAS). A two-way analysis of variance evaluated the inoculum (Control vs. stimbiotic) and treatment (Control, XYL, XOS, sDP-XOS, lDP-XOS and WB) as main effects on gas production, SCFA, microbiota and disappearance of carbohydrates. The simulation vessel was considered a random effect. Means were separated by Student’s t-test. Statements of significance were based on *p*-value of equal or less than 0.05.

## 3 Results

### 3.1 Total gas production

**Figure 2:**
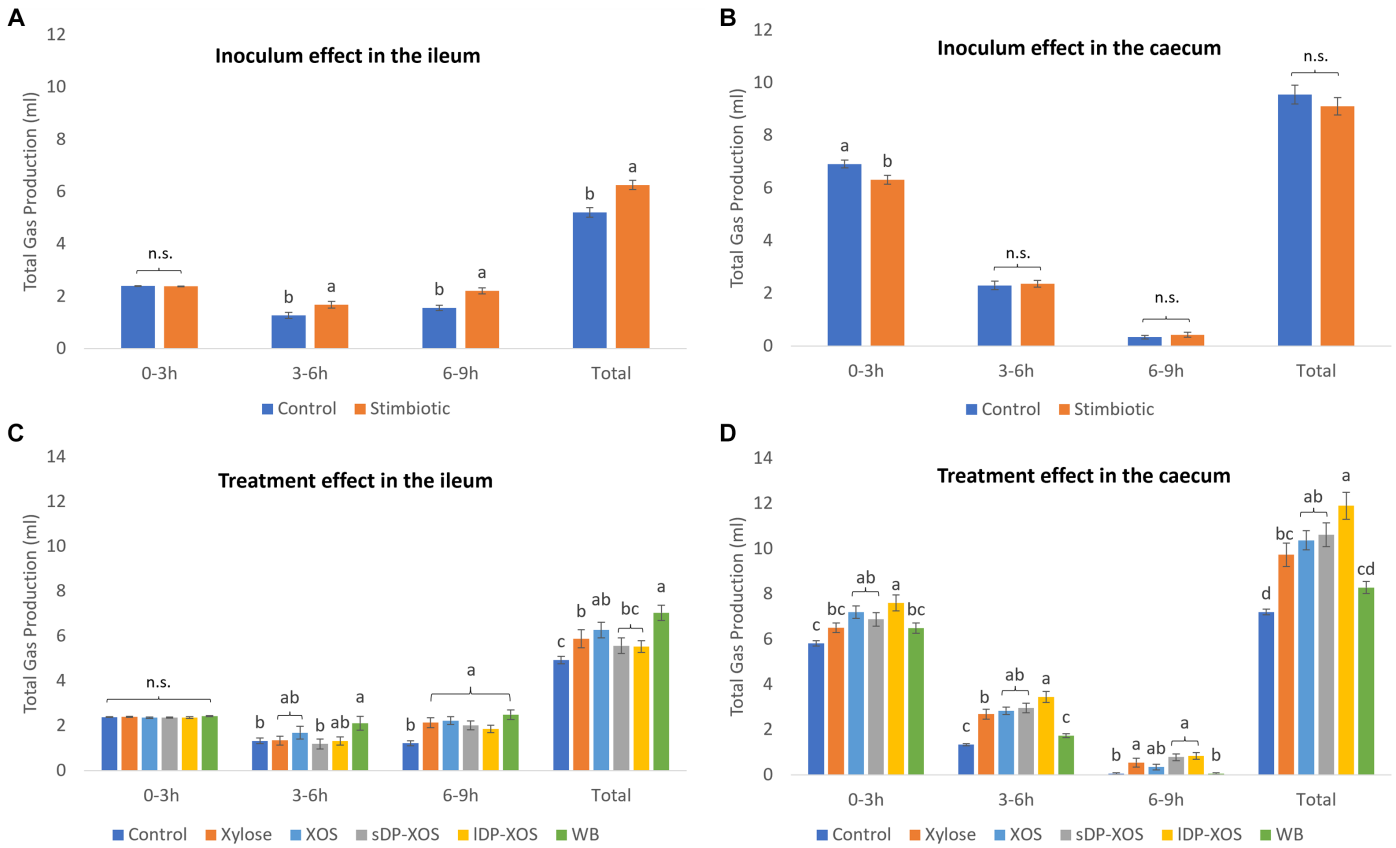
Gas produced (ml) during *ex vivo* fermentation (*n* = 6 for each treatment, n = 12 for the blank control). Inoculum effect on gas production in the ileum **(A)** and caecum **(B)**. Treatment effect on gas production in the ileum **(C)** and caecum **(D)**. Columns are grouped into different lengths of fermentation in hours. Significant differences (*p* < 0.05) within each time period are shown as different letters above each column. Bars indicate standard error of the mean.

No interactions were observed for total gas production in either the ileum or the caecum and therefore, results of the main factors are shown in Figure 2. In general, in the ileal *ex vivo* simulation, gas was produced steadily over the 9 h fermentation period (Figure 2A). Ileal microbiota from stimbiotic birds produced more gas by the end of the 9 h incubation (*p* < 0.001) compared to the control, especially driven by the increased fermentation activity observed from 3 h to 6 h (*p* < 0.05) and 6 h to 9 h (*p* < 0.05). The different xylose-rich carbohydrate substrates did not influence gas production in the first 3 h of incubation (Figure 2C). Substrates, however, did influence the cumulative total gas production after the full 9 h incubation (*p* < 0.001). Ileal microbiota growing on WB were producing significantly (*p* < 0.05) more gas, followed by the unfractionated XOS and monomeric XYL, compared to the blank control treatment.

In the caecal *ex vivo* simulation, the greatest amount of gas was produced in the first 3 h of fermentation with longer periods of incubation resulting in a diminishing rate of gas production (Figure 2B). Caecal innocula from stimbiotic birds showed lower gas production in the first 3 h of incubation compared to innocula from control birds (*p* < 0.001). The different xylose-rich carbohydrate substrates significantly influenced gas production in all time ranges (Figure 2D) (*p* < 0.001). All vessels provided with a carbohydrate substrate, except for WB, produced significantly more gas than the blank control. The greatest amount of gas was produced by caecal microbiota exposed to XOS substrates, in particular the lDP-XOS, followed by sDP-XOS, XOS and monomeric XYL.

### 3.2 Fermentation end-products

No interactions were observed on the fermentation end-products after 9 h of incubation in the *ex vivo* experiment either with ileal or caecal innocula. The main effects are presented in Table 3. In the ileal simulation, supplementing diets with the stimbiotic resulted in higher VFA concentrations (*p* < 0.0001), but no differences were observed in the lactic acid concentration or the ratio between lactic acid and acetic acid (*p* > 0.05). All the xylose-rich substrates resulted in greater production of VFAs compared to the blank control (*p* < 0.001), but there was no significant difference between them. Lactic acid levels were highest when the ileal digesta was exposed to WB.

**Table 3:** Interactive effects of inoculum and substrate on fermentation end-products after 9 h of incubation in the *ex vivo* assay with ileal and caecal digesta.

			VFA	Lactic acid	Lactic:Acetic	VFA:BCFA
Ileum						
Inoculum						
		Control	8.8 ± 1.14^b^	22.4 ± 5.40	2.7 ± 0.93	ND
		Stimbiotic	9.9 ± 1.14^a^	22.9 ± 5.40	2.4 ± 0.93	ND
Treatment						
		Control	7.9 ± 1.11^b^	20.2 ± 5.22^b^	2.7 ± 0.90	ND
		Xylose	10.0 ± 1.11^a^	19.5 ± 5.22^b^	2.0 ± 0.90	ND
		XOS	9.4 ± 1.11^a^	21.8 ± 5.22^b^	2.4 ± 0.90	ND
		sDP XOS	10.2 ± 1.11^a^	24.3 ± 5.22^ab^	2.5 ± 0.90	ND
		lDP XOS	9.2 ± 1.11^a^	21.8 ± 5.22^b^	2.5 ± 0.90	ND
		Wheat bran	9.6 ± 1.11^a^	28.5 ± 5.22^a^	3.1 ± 0.90	ND
*p*-Value						
		Inoculum	<0.001	0.644	0.160	–
		Treatment	<0.001	<0.001	0.089	–
		Interaction	0.930	0.936	0.993	–
Caecum						
Inoculum						
		Control	60.3 ± 5.30^a^	2.0 ± 3.24^b^	0.04 ± 0.08^b^	143 ± 28^b^
		Stimbiotic	56.5 ± 5.30^b^	3.6 ± 3.24^a^	0.09 ± 0.08^a^	156 ± 28^a^
Treatment						
		Control	44.8 ± 5.12^d^	1.2 ± 3.13^b^	0.04 ± 0.08^b^	70 ± 27^c^
		Xylose	68.3 ± 5.13^a^	1.7 ± 3.13^b^	0.03 ± 0.08^b^	158 ± 27^b^
		XOS	60.5 ± 5.12^b^	2.3 ± 3.13^b^	0.06 ± 0.08^ab^	183 ± 27^ab^
		sDP XOS	62.9 ± 5.12^ab^	3.8 ± 3.13^ab^	0.09 ± 0.08^ab^	209 ± 27^a^
		lDP XOS	63.6 ± 5.12^ab^	7.1 ± 3.13^a^	0.15 ± 0.08^a^	201 ± 27^a^
		Wheat bran	50.6 ± 5.12^c^	0.8 ± 3.13^b^	0.02 ± 0.08^b^	74 ± 27^c^
*p*-value						
		Inoculum	0.002	0.027	0.020	0.034
		Treatment	<0.001	<0.001	0.001	<0.001
		Interaction	0.702	0.823	0.865	0.966

The values are reported in mmol/L as the mean ± standard deviation of 6 replicates for each substrate treatment and 12 replicates for the blank control. VFAs, volatile fatty acids; BCFA, branched-chain fatty acids. ^a-c^Means with a different superscript letter differ (*p* < 0.05). ND, non-detected.

In the caecal simulation, the stimbiotic inoculum resulted in lower VFA concentrations (*p* < 0.01), higher lactic acid levels (*p* < 0.05), a higher lactic acid to acetic acid ratio (*p* < 0.05), and a higher VFA to BCFA ratio (*p* < 0.05). Substrates also influenced VFA (*p* < 0.001), lactic acid (p < 0.001) and the ratios of lactic acid to acetic acid (*p* = 0.001) and VFA to BCFA (*p* < 0.001). WB appeared to be a poor fermentation substrate for caecal microbes showing VFA concentrations similar to the control. In contrast, XYL > lDP-XOS > sDP-XOS > XOS presented higher VFA concentrations compared to WB and the control. lDP-XOS showed the highest caecal lactic acid concentration and lactic acid to acetic acid ratio compared to all the other substrates. Both XOS fractions, sDP-XOS and lDP-XOS, presented the highest VFA to BCFA ratio followed by the original XOS and monomeric XYL. WB did not influence the VFA to BCFA ratio compared to control (*p* > 0.05).

### 3.3 Ileal bacteria

No interactions or substrate effects were observed on microbiota after 9 h of incubation in the *ex vivo* assay with ileal digesta (Table 4). Ileal inoculum from stimbiotic birds showed higher counts of total eubacteria (*p* < 0.001), *Escherichia coli* (*p* < 0.001), *Lactobacillus* spp. (*p* < 0.001), *L. salivarius* (*p* < 0.001) and *L. crispatus* (*p* < 0.001). Lower *Enterococcus* spp. counts were observed in stimbiotic inocula after 9 h of incubation (*p* < 0.01). *Streptococcus* spp. and *L. reuteri* were not influenced by inoculum (*p* > 0.05).

**Table 4:** Interactive effects of inoculum and substrate on microbiota after 9 h of incubation in the *ex vivo* assay with ileal digesta.

		Total eubacteria	*Streptococcus* spp	*Enterococcus* spp	*Escherichia coli*	*Lactobacillus* spp	*L. salivarius*	*L. reuteri*	*L. crispatus*
Inoculum									
	Control	9.63 ± 0.21^b^	8.39 ± 0.51	8.59 ± 0.25^a^	9.10 ± 0.17^b^	8.65 ± 0.47^b^	6.43 ± 0.70^b^	8.06 ± 0.63	8.61 ± 0.40^b^
	Stimbiotic	9.90 ± 0.22^a^	8.51 ± 0.51	8.43 ± 0.25^b^	9.36 ± 0.18^a^	9.23 ± 0.48^a^	7.65 ± 0.71^a^	8.06 ± 0.64	8.95 ± 0.40^a^
Treatment									
	Control	9.73 ± 0.22	8.39 ± 0.51	8.48 ± 0.25	9.17 ± 0.17	8.91 ± 0.47	7.02 ± 0.71	7.97 ± 0.63	8.66 ± 0.40
	Xylose	9.84 ± 0.21	8.52 ± 0.49	8.53 ± 0.24	9.31 ± 0.17	8.85 ± 0.45	7.12 ± 0.68	7.96 ± 0.60	8.66 ± 0.38
	XOS	9.74 ± 0.21	8.30 ± 0.49	8.47 ± 0.24	9.16 ± 0.17	8.89 ± 0.45	6.88 ± 0.68	8.16 ± 0.60	8.81 ± 0.38
	sDP XOS	9.63 ± 0.21	8.42 ± 0.49	8.44 ± 0.24	9.15 ± 0.17	8.83 ± 0.45	6.78 ± 0.68	7.93 ± 0.61	8.77 ± 0.38
	lDP XOS	9.74 ± 0.21	8.42 ± 0.50	8.54 ± 0.24	9.26 ± 0.17	8.93 ± 0.46	7.07 ± 0.69	7.95 ± 0.62	8.74 ± 0.39
	Wheat bran	9.89 ± 0.21	8.65 ± 0.49	8.60 ± 0.24	9.30 ± 0.17	9.23 ± 0.45	7.39 ± 0.68	8.38 ± 0.61	9.03 ± 0.39
*p*-value									
	Inoculum	<0.001	0.301	0.006	<0.001	<0.001	<0.001	0.979	<0.001
	Treatment	0.083	0.659	0.667	0.053	0.376	0.421	0.478	0.217
	Interaction	0.436	0.961	0.814	0.673	0.657	0.914	0.863	0.641

The values are reported as the mean ± standard deviation in log10 gene copies/ml produced as the mean of 6 replicates for each substrate treatment and 12 replicates for the blank control. ^a-b^Means with a different superscript letter differ (*p* < 0.05).

### 3.4 Carbohydrates

To evaluate the effect of the stimbiotic on the ability of the ileal microbial community to degrade the different xylose-rich carbohydrate substrates, the total carbohydrate content from the inoculum and substrates was measured before and after 9 h of incubation. The percentage loss of the different sugars are presented in Table 5. No interactions were observed. The inoculum from the stimbiotic-supplemented birds did not influence the loss of xylose, arabinose, mannose and glucose over the 9 h of the *ex vivo* experiment, however 2% more galactose disappeared compared to control inoculum (*p* < 0.05). The type of substrate had a more significant impact on the utilisation of sugars. Monomeric XYL and all XOS substrates decreased the utilisation of xylose compared to the control (*p* < 0.05), while no differences were observed between control and WB. Ileal arabinose disappearance was higher with all the substrates compared to the control, with sDP-XOS presenting the highest value, followed by XOS, lDP-XOS and WB, and XYL. XYL as a substrate did not influence mannose fermentation, in contrast all sources of XOS and WB increased its utilisation. Although substrate influenced the fermentation of galactose (*p* = 0.002), none of the products differed significantly from the control. No differences were observed in glucose disappearance with XYL, XOS, or sDP-XOS compared to the control, but lDP-XOS showed lower utilisation rate while WB clearly had higher utilisation.

**Table 5:** Interactive effects of inoculum and substrate on disappeared total soluble sugars after 9 h of incubation in the *ex vivo* assay with ileal digesta.

		Xylose	Arabinose	Mannose	Galactose	Glucose
Inoculum						
	Control	34.3 ± 4.33	49.8 ± 2.37	47.0 ± 1.48	56.3 ± 2.74 ^b^	79.1 ± 1.11
	Stimbiotic	34.2 ± 4.33	48.8 ± 2.37	46.5 ± 1.48	58.2 ± 2.74 ^a^	80.5 ± 1.11
Treatment						
	Control	45.1 ± 4.18^a^	43.6 ± 2.29^d^	35.5 ± 1.44^c^	57.3 ± 2.65^ab^	80.2 ± 1.07^b^
	Xylose	21.3 ± 4.18^c^	47.3 ± 2.29^c^	36.4 ± 1.44^c^	56.2 ± 2.65^b^	79.7 ± 1.07^b^
	XOS	32.5 ± 4.18^b^	52.1 ± 2.29^b^	52.7 ± 1.44^a^	57.9 ± 2.65^ab^	79.6 ± 1.07^b^
	sDP XOS	32.5 ± 4.18^b^	55.6 ± 2.29^a^	53.3 ± 1.44^a^	57.0 ± 2.65^ab^	80.2 ± 1.07^b^
	lDP XOS	25.4 ± 4.18^c^	48.6 ± 2.29^c^	50.6 ± 1.44^b^	55.2 ± 2.65^b^	74.2 ± 1.07^c^
	Wheat bran	48.6 ± 4.18^a^	48.6 ± 2.29^c^	51.9 ± 1.44^ab^	59.8 ± 2.65^a^	82.0 ± 1.07^a^
*p*-value						
	Inoculum	0.903	0.074	0.111	0.002	0.126
	Treatment	<0.001	<0.001	<0.001	0.002	<0.001
	Interaction	0.645	0.980	0.681	0.984	0.782

The values reported are the mean % ± standard deviation of total soluble sugars loss of 6 replicates for each substrate treatment and 12 replicates for the blank control. ^a-d^Means with a different superscript letter differ (*p* < 0.05).

## 4 Discussion

The aim of the current study was to investigate the reaction of the ileal and caecal microbiota from control or stimbiotic-supplemented birds when exposed to different xylose-rich carbohydrate substrates in an *ex vivo* experiment by measuring total gas production, fermentation end-products, bacterial counts, and loss of carbohydrates after 9 h of incubation. No interactions were observed in any of the parameters measured suggesting that the response of the bacterial communities to the different substrates provided was independent of the stimbiotic treatment for both intestinal compartments. Thus, the first hypothesis provided in this study was not confirmed. There may be several reasons for the lack of interactive effects. Firstly, bird age can have a significant effect on microbiota composition (Gong et al., 2008). In young birds, the gut microbiome is less well developed and more likely to be influenced by dietary changes, whereas older birds can efficiently utilise intransigent nutrients like dietary fibre (Lee et al., 2017; Craig et al., 2020). At 4 weeks old, the birds in this study should have had time to develop a robust gut microbiome which may influence the response in the *ex vivo* experiment. Secondly, caecal emptying occurs several times a day in chickens. A recently emptied caecum can have a very different microbial composition to one that is full (Richards et al., 2019). Therefore, the timing of when inocula are taken adds another source of variation on top of the individual variation between birds. Thirdly, the concentration of the xylose-rich substrates used in the vessels was above the doses normally used *in vivo*. This may have led to saturation in the assay. Morgan et al. (2022) also observed that stimulating fibre-degrading bacteria with high levels of XOS in diets with little fermentable arabinoxylan can result in detrimental effects on the microbial balance and a proliferation of pathogenic bacteria species which outcompete the beneficial bacteria. Although no interactions were observed, the main effects are discussed in the next sections.

### 4.1 Effects of stimbiotic on the ileal and caecal microbial activity

This study found evidence that feeding broiler chickens a stimbiotic can modulate the microbiome and improve its fibre fermenting capabilities, however there were clear differences between the ileal and caecal microbial responses.

The stimbiotic effect stipulates that small doses of dietary additives can stimulate a fibre-degrading microbiome to increase fibre fermentability (González-Ortiz et al., 2019). In the literature, supplemental XOS doses range from as low as 2 g/t to 20,000 g/t, 50 g/t being the most tested dose across different studies showing positive effects (Morgan et al., 2023). The present study found evidence for this effect in the ileum where microbiota derived from birds fed a stimbiotic were significantly more active as demonstrated by higher gas production, higher VFA concentrations, higher abundances of total eubacteria driven primarily by *Lactobacillus* spp., and greater utilisation of galactose. Thus, the second hypothesis in this study was supported. The stimbiotic in this study was a combination of xylanase and XOS. Both products have been demonstrated to positively influence cumulative bird performance through enhanced non-starch polysaccharide utilisation resulting in the proliferation of beneficial bacteria and SCFA production (González-Ortiz et al., 2021; Morgan et al., 2021; Singh et al., 2021). These effects are also supported by a recent holistic evaluation, which has identified that supplementing XOS into broiler diets reduces bird mortality and improves FCR (Morgan et al., 2023).

The ileal microbial community is mainly comprised of lactic acid producing bacteria (Rinttilä and Apajalahti, 2013). After 9 h of incubation in the *ex vivo* model, the lactic acid producing bacterial species *L. salivarius* and *L. crispatus* were both more abundant in the stimbiotic treatments. This is consistent with previous studies on swine where supplementation with XOS results in higher lactobacilli abundance in ileal, caecal, and faecal samples (Liu et al., 2018; Pan et al., 2019; Chen et al., 2021). In one *ex vivo* study, Moura et al. (2008) showed that when ileal inocula from piglets were provided with a XOS substrate, bifidobacteria and lactobacilli replication was enhanced. The primary metabolite of lactobacilli is lactic acid. Lactic acid plays a major role in lowing the pH of the ileum and preventing colonization by pH-sensitive pathogenic bacteria (Pan et al., 2019). This was demonstrated in the current study by the lower abundance of *Enterococcus* spp. in stimbiotic treatments. Despite higher lactobacilli abundance in stimbiotic treatments, this study found no significant increase in lactic acid in the ileum fermentation model. This could be because lactic acid is an intermediary product that can be further metabolized into VFAs such as acetate, propionate and butyrate by other common intestinal microbes. This type of cross-feeding between bacteria has been observed *in vitro* by De Maesschalck et al. (2015) where lactic acid produced by *L. crispatus* during XOS fermentation was utilised by the butyrate-producing species *Anaerostipes butyraticus*. This explanation is consistent with the observation that although lactic acid concentration was unchanged, there were overall significantly higher concentrations of VFAs in stimbiotic treatments.

Xylanases are typically associated with the hydrolysis of arabinoxylans, however increased levels of fructose and galactose have also been observed *in vivo* after the addition of xylanase (Craig et al., 2020). Galactose likely originates from galactooligosaccharides or galactans which are found in soybean meal, a common protein source in poultry feed. The increased fermentation of galactose in stimbiotic treatments observed in this study could be due to the *in situ* release of galactose through xylanase action.

Caecal microbiota from stimbiotic-supplemented birds were less active in terms of gas and VFA production. Gas production was highest in the first 3 h of fermentation and then the rate of production declined, which may suggest a limiting resource that has been exhausted more rapidly by the stimbiotic effect. However, it is interesting to highlight the higher lactic acid concentrations, higher lactic acid:acetic acid ratio, and higher VFA:BCFA ratio promoted with the stimbiotic caecal inoculum. The increased lactic acid concentrations and higher lactic acid:acetic acid ratio may indicate the promotion of homofermentive lactic acid producing bacterial species over heterofermenters in the caecum by the stimbiotic treatment. The higher VFA:BCFA ratio (up to 9%) promoted with the stimbiotic demonstrates how the stimbiotic effect can reduce protein fermentation by providing caecal bacteria with carbohydrates, which are preferentially fermented over proteins (Xu et al., 2002; Sanchez et al., 2009). This can be beneficial for the host since increased caecal BCFA production may imply the concomitant production of toxic fermentation products such as ammonia and biogenic amines, which can inhibit growth and cause disease (Apajalahti and Vienola, 2016). Similar reduction of lower intestinal protein fermentation has been observed in piglets supplemented with the stimbiotic (Cho et al., 2020), and broiler chickens supplemented with a xylanase (Lee et al., 2017).

### 4.2 Effects of xylose-rich carbohydrate substrates on the ileal and caecal microbial activity

When comparing the different xylose-rich carbohydrate substrates, ileal microbiota showed a distinct preference for WB, which is observed by the greatest gas production and highest lactic acid concentrations. Previous *in vitro* studies using faecal inocula have similarly found that the amount of gas produced by microbiota varies depending on the substrate it is fermenting (Tao et al., 2019). Typically, longer chain oligosaccharides and more complex polysaccharides are less readily fermented and produce less gas (Moura et al., 2008; Hernot et al., 2009; Kaur et al., 2011). However, this study found no evidence in the gas production, fermentation end-products, or bacterial community, that shorter oligosaccharides are more readily fermented by ileal microbiota than longer ones. Thus, the third hypothesis in this study was not confirmed. It is possible that despite being the most complex substrate, wheat bran was preferred due to its sugar composition. Wheat bran had the lowest xylose content of the substrates used in this study and had proportionally higher glucose content. Across all treatments, glucose was the most fermented sugar (74–82% loss), while xylose was the least fermented (21–48% loss), suggesting a microbial preference for glucose. In addition, some studies have shown that the availability of glucose can delay the utilisation of lactic acid by lactic acid utilising bacteria resulting in lactic-acid accumulation (Duncan et al., 2004).

In contrast to the ileal results, wheat bran appeared to be a poor fermentation substrate for caecal microbiota. Wheat bran had the lowest gas production of all the substrates and was indistinguishable from the blank control in terms of statistical significance. However, all XOS-derived substrates promoted caecal microbial activity. In particular, lDP-XOS had the highest gas production, lactic acid concentration, and lactic to acetic acid ratio. Bacteria species vary significantly in their ability to use XOS of differing chain lengths depending on their ability to produce extra-and intracellular hydrolytic enzymes, as well as mono-and oligosaccharide transporters involved in carbohydrate metabolism (Rastall et al., 2022). This can make it difficult to predict how the diverse caecal microbiome will respond to XOS of different lengths. Wang et al. (2010) observed that *Bifidobacterium adolescentis* utilised DP3 and DP2 more rapidly than monosaccharides or DP4-7, while Dale et al. (2020) found evidence that caecal bacteria in broilers completely utilised DP4, but DP3 and DP2 were not fully utilised. The preference for higher DP XOS seen in this study suggests that longer chain oligosaccharides may be promising candidates for future products that can reach and be fermented in distal sections of the intestinal tract.

## 5. Conclusion

The *ex vivo* approach is a valuable tool for studying the impact of feed additives on microbial communities. The functional response of entire intestinal microbial communities can be studied in a more controlled way than in a live host environment. This study indicates that supplementing broiler diets with a stimbiotic promotes the abundance of lactic acid producing bacteria and SCFA content in the gut which could have beneficial effects on broiler performance. Ileal bacteria favoured glucose-rich wheat bran over XOS substrates, while caecal bacteria appeared to favour long-chain xylooligosaccharides. Against predictions, there was no indication that microbiota from stimbiotic birds were primed to favour XOS substrates when compared to microbiota from control birds. This study highlights some of the mechanisms by which stimbiotics can potentially impact broiler performance through microbiome modulation and demonstrates a methodology to identify promising future stimbiotic candidates.

## Data availability statement

The raw data supporting the conclusions of this article will be made available by the authors, without undue reservation.

## Author contributions

CD: Writing – original draft, Data curation, Formal analysis, Funding acquisition, Investigation, Methodology, Project administration, Resources, Supervision, Validation, Visualization. GG-O: Conceptualization, Data curation, Formal analysis, Funding acquisition, Investigation, Methodology, Project administration, Resources, Supervision, Validation, Visualization, Writing – original draft. TR: Conceptualization, Data curation, Formal analysis, Funding acquisition, Investigation, Methodology, Project administration, Resources, Supervision, Validation, Visualization, Writing – original draft. JA: Methodology, Conceptualization, Data curation, Funding acquisition, Investigation, Project administration, Supervision, Validation, Writing – review & editing. MA: Methodology, Resources, Writing – original draft. MB: Conceptualization, Data curation, Funding acquisition, Investigation, Methodology, Project administration, Resources, Supervision, Validation, Writing – review & editing.

## Ethics statement

The animal study was approved by The Regional Administrative Agency of Southern Finland (ESAVI). The study was conducted in accordance with the local legislation and institutional requirements.
