# Supplementary material for: Stimbiotic supplementation and xylose-rich carbohydrates modulate broiler’s capacity to ferment fibre
Source: Front Microbiol. 2024 Jan 11;14:1301727. doi: 10.3389/fmicb.2023.1301727 (PMC10808361; doi:10.3389/fmicb.2023.1301727)
Supplement: Supplementary file 1 [file Table_1.DOCX]

**Supplementary Table 1.** Broiler diet.

Starter % Grower %

(d 0-21) (d 21-42)

Wheat 65.79 73.44

Soybean meal (Hipro) 28.69 20.98

Soy oil 2.73 3.45

Monocalcium phosphate 0.59 0.09

Limestone 0.97 0.84

NaCl 0.32 0.32

Mineral premix* 0.25 0.25

Vitamin premix** 0.25 0.25

DL-Methionine 0.19 0.15

L-Lysine HCl 0.18 0.18

Threonine 0.03 0.04

Quantum blue 5G 0.01 0.01

Total 100 100

*) calcium 296.9 g/kg, zinc 32.5 g/kg, manganese 25.0 g/kg, iron 12.5 g/kg, copper 4.0 g/kg, iodine 225 mg/kg, selenium 100 mg/kg.

**) Contents of the vitamin premix: calcium 331.3 g/kg, all-rac-α-tocopheryl acetate 30.0 g/kg, niacin 20.1 g/kg, panthotenic acid 7.51 g/kg, riboflavin 3.0 g/kg, pyridoxine 2.01 g/kg, retinol 1.8 g/kg, menadione 1505 mg/kg, thiamine 1257 mg/kg, folic acid 504 mg/kg, biotin 75.0 mg/kg, cholecalciferol, 56.3 mg/kg, cobalamin 12.5 mg/kg
