# Supplementary material for: Stimbiotic supplementation and xylose-rich carbohydrates modulate broiler’s capacity to ferment fibre
Source: Front Microbiol. 2024 Jan 11;14:1301727. doi: 10.3389/fmicb.2023.1301727 (PMC10808361; doi:10.3389/fmicb.2023.1301727)
Supplement: Supplementary file 2 [file Data_Sheet_1.docx]

**Supplementary Figure 1.** Amplification, standard and melt curves of (a) *L. salivarius*, (b) *L. reuteri*, and (c) *L. crispatus*, (d) *Enterococcus* spp. and (e) *E. coli* targeted assays. The standard curves of SYBR Green I assays were obtained by plotting the threshold cycle values against the target DNA starting quantity. Synthetic standard DNA of the target species was used as the template in quantities from 1 × 10^8^ to 1 × 10^2^ per PCR reaction.

1. ***L. salivarius***


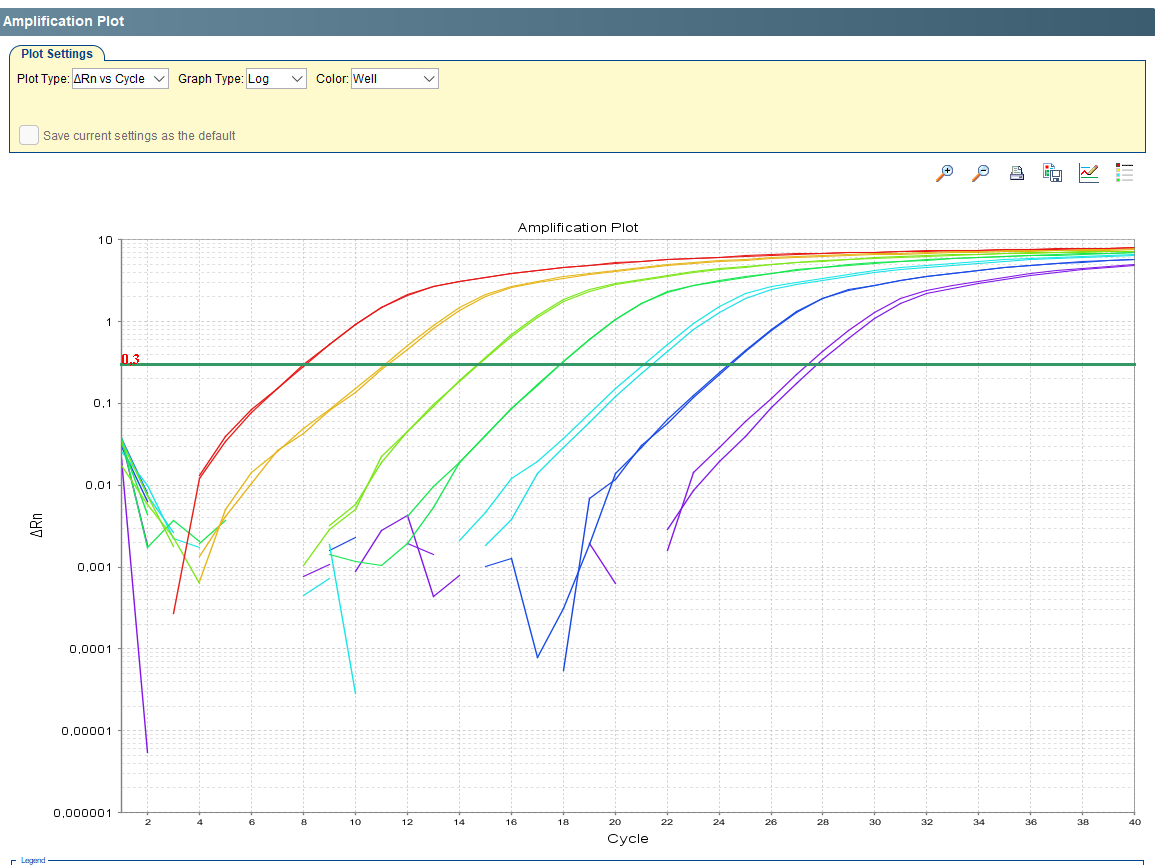


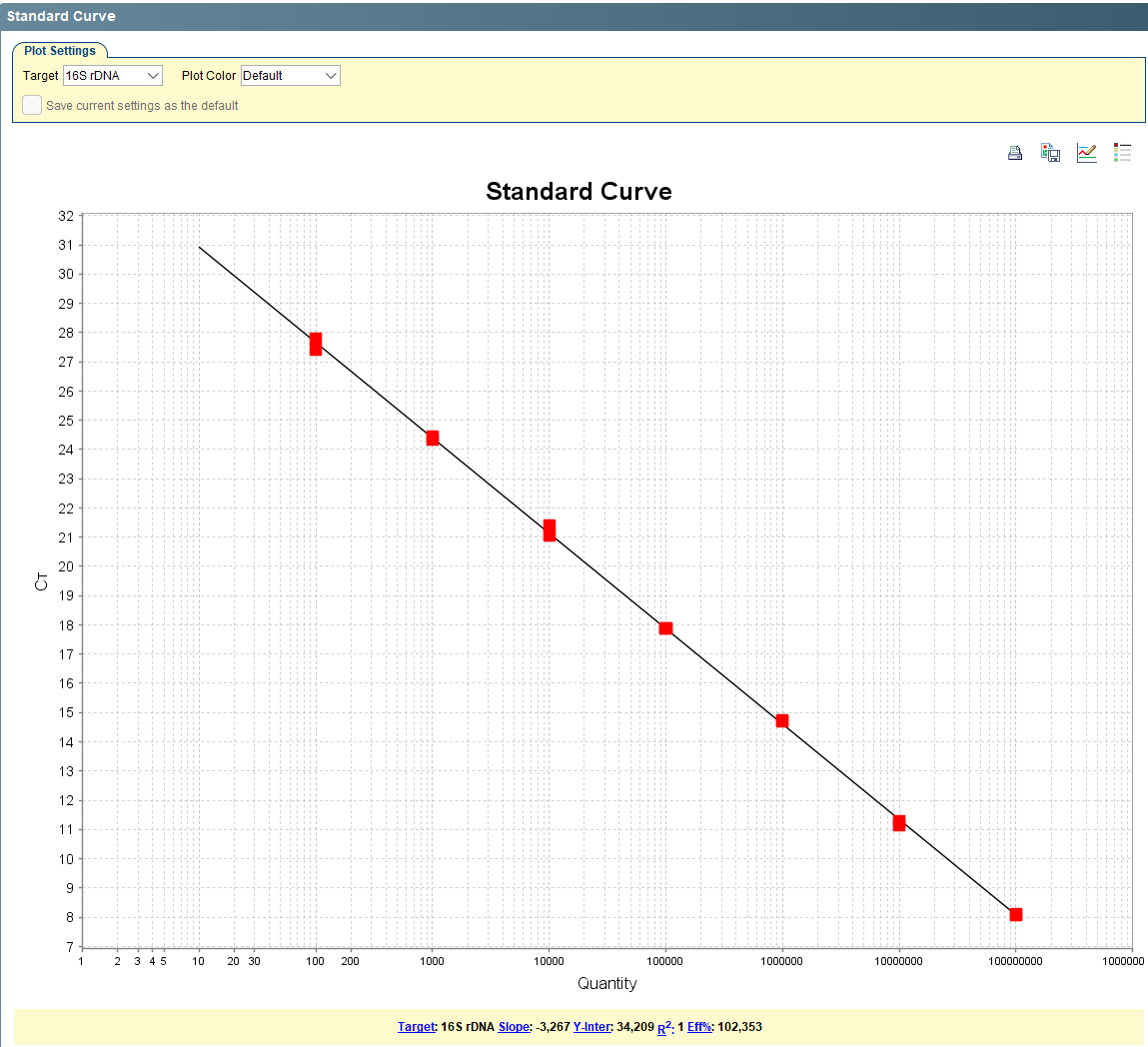


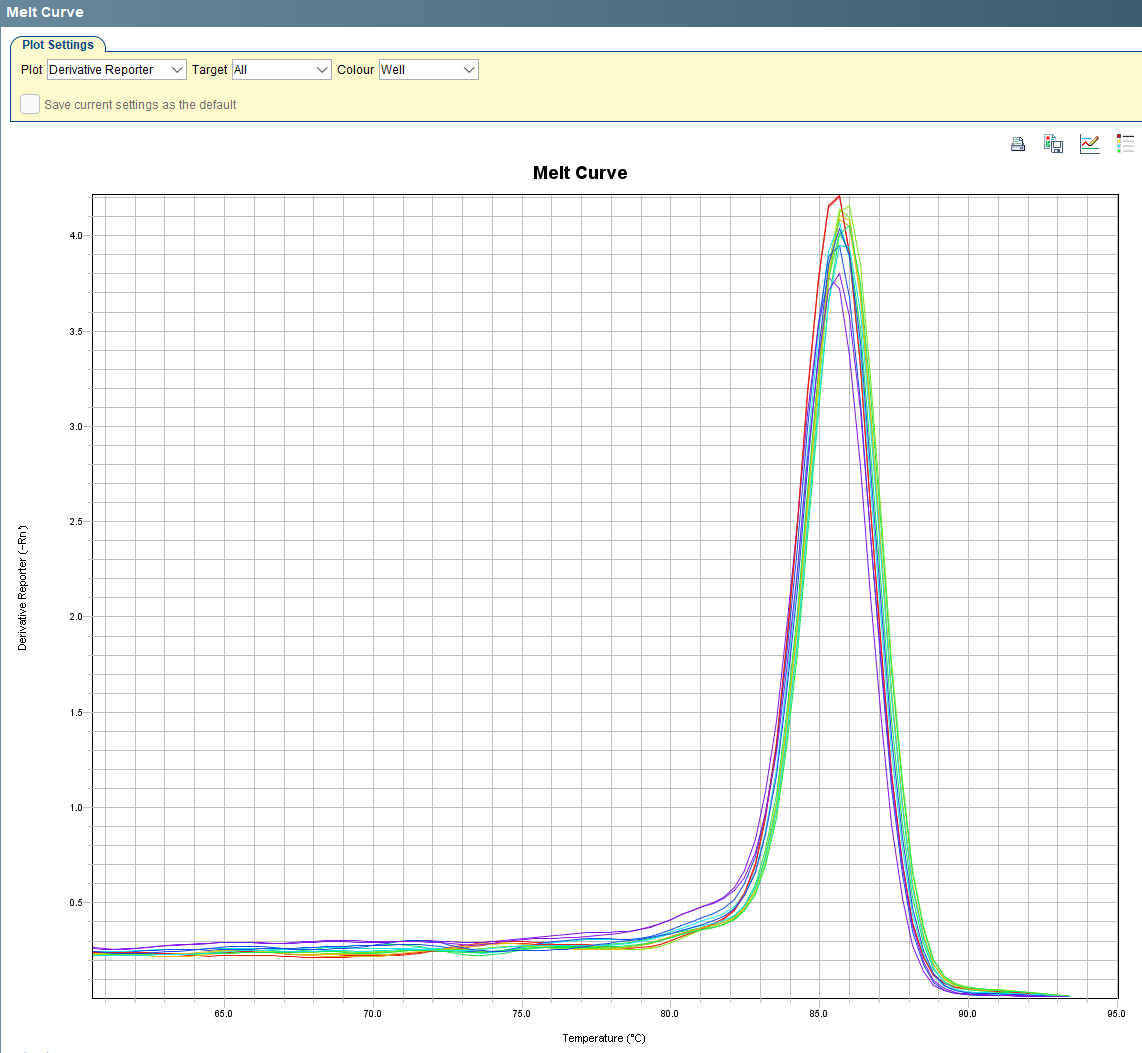


1. ***L. reuteri***


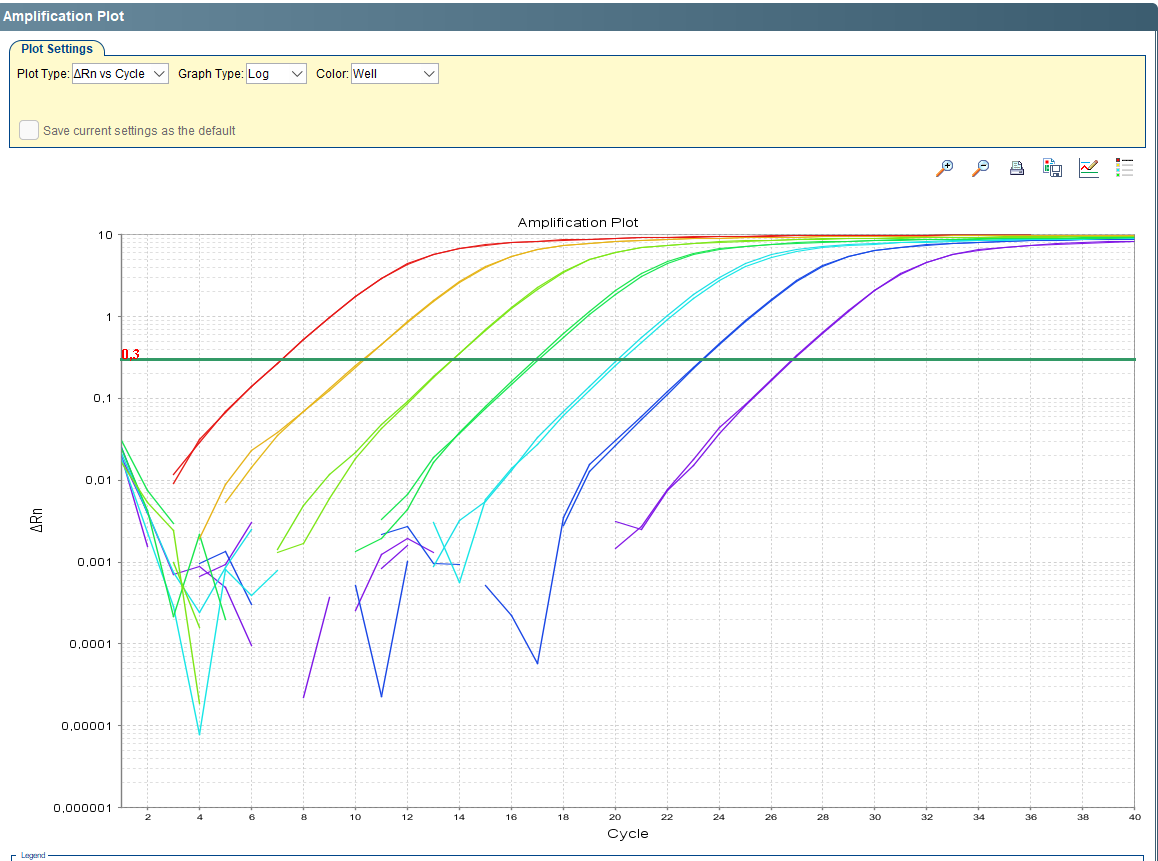


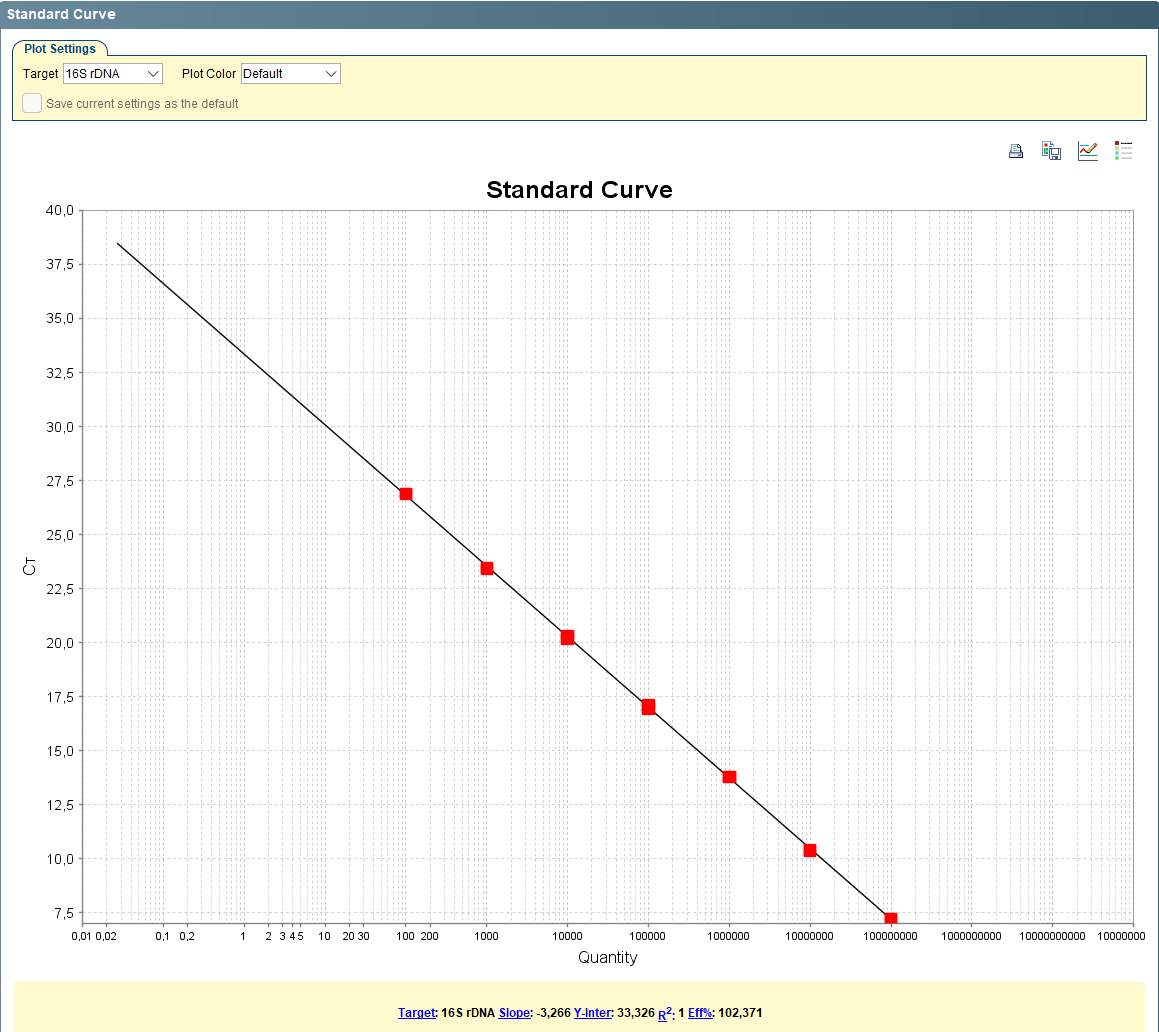


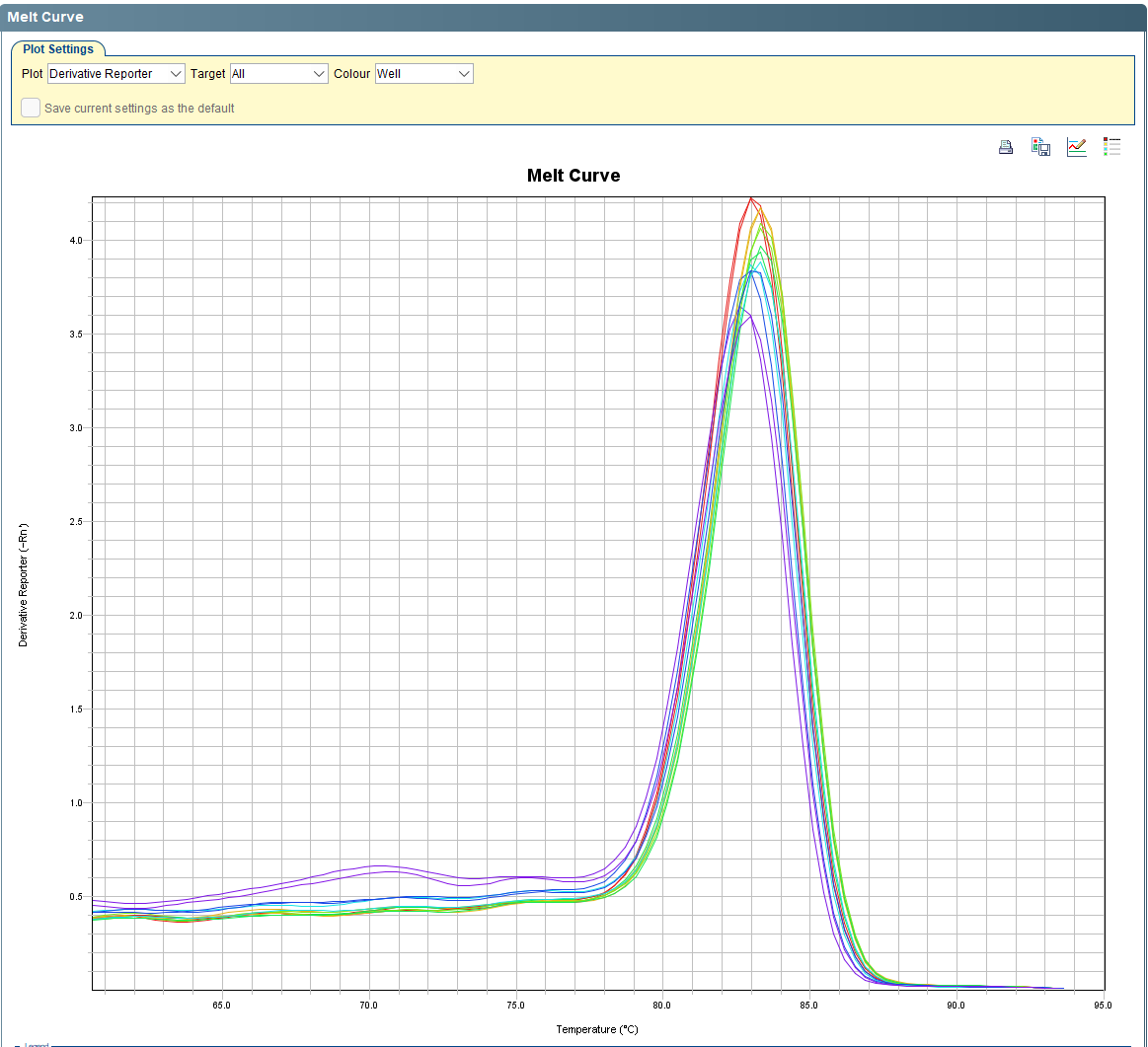


***L. crispatus***


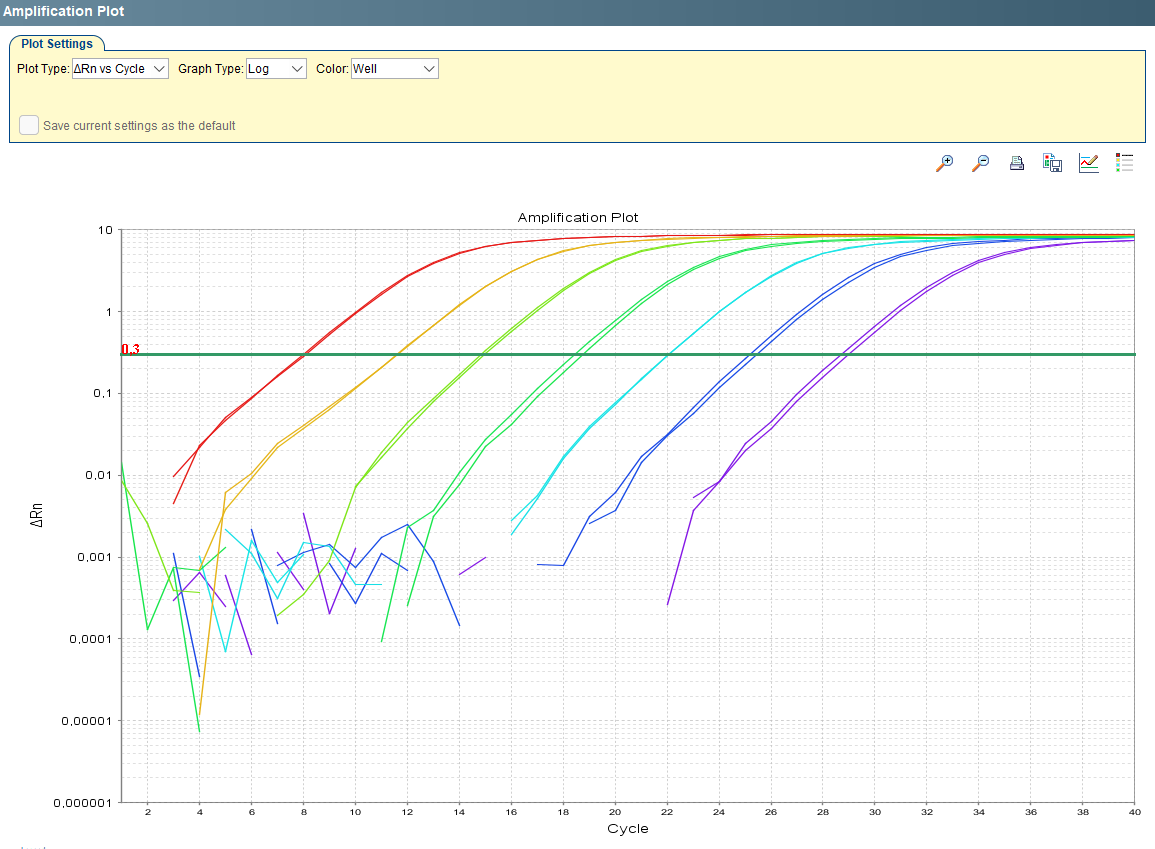


**
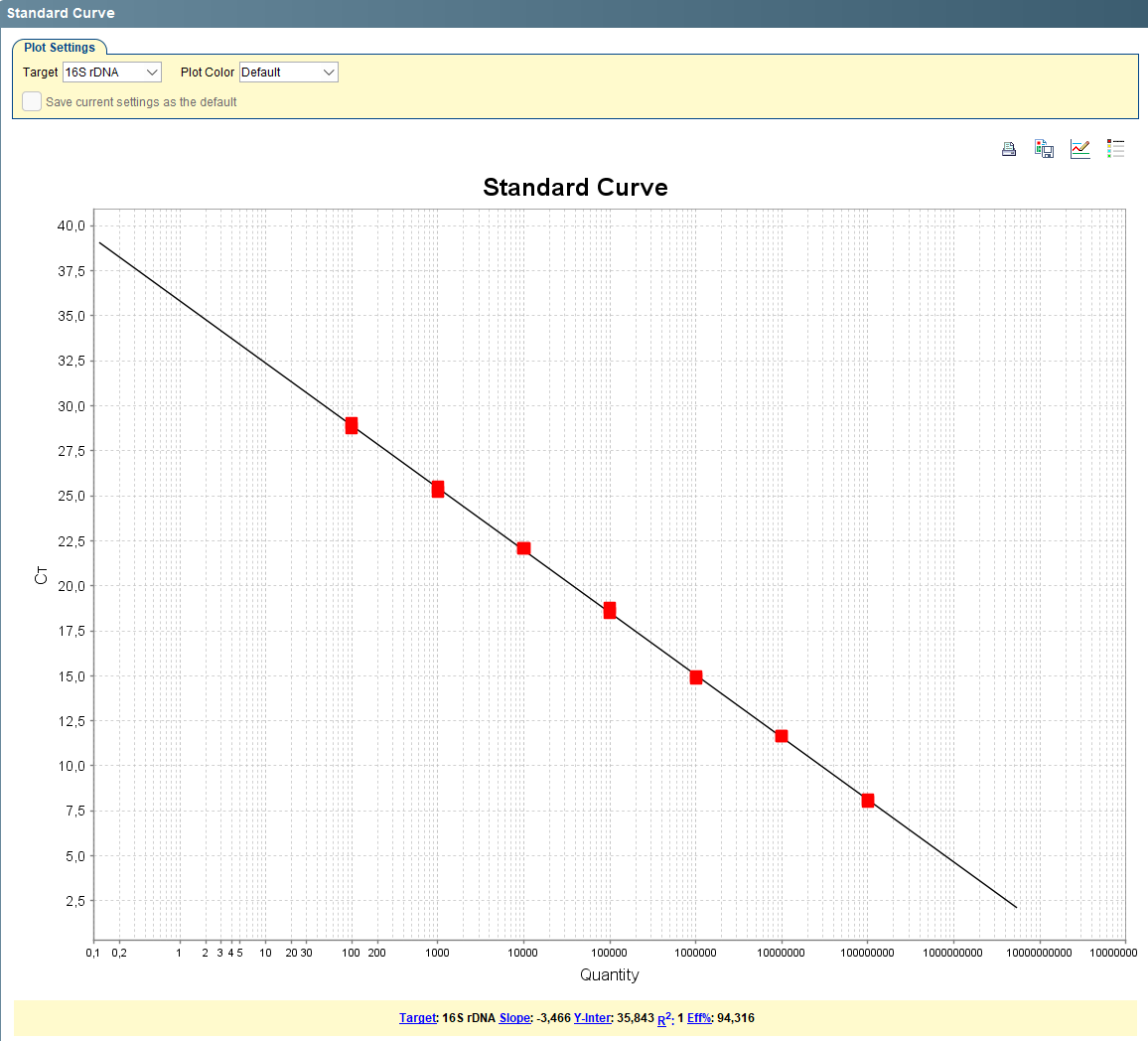
**


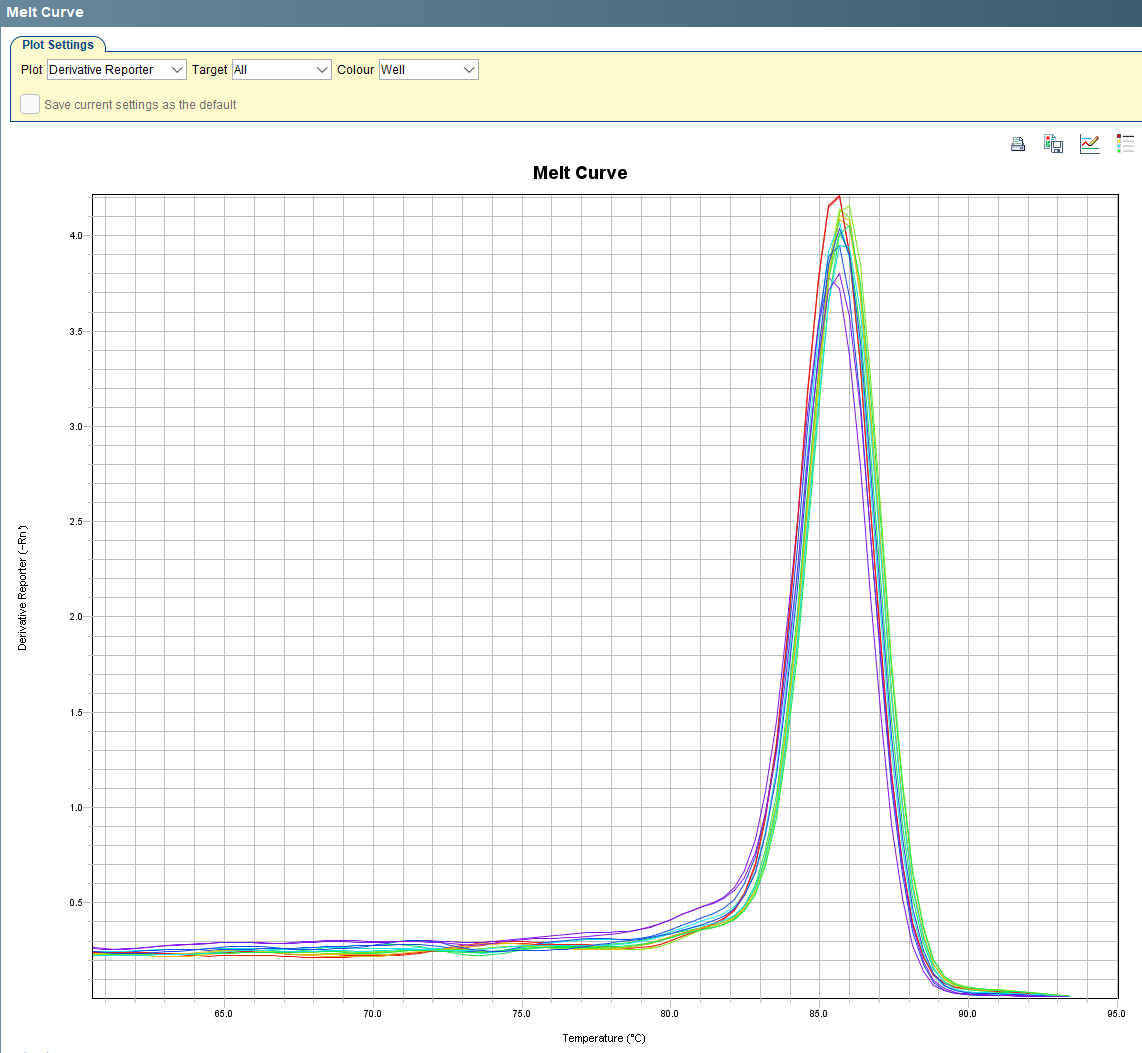


1. ***Enterococcus* spp.**


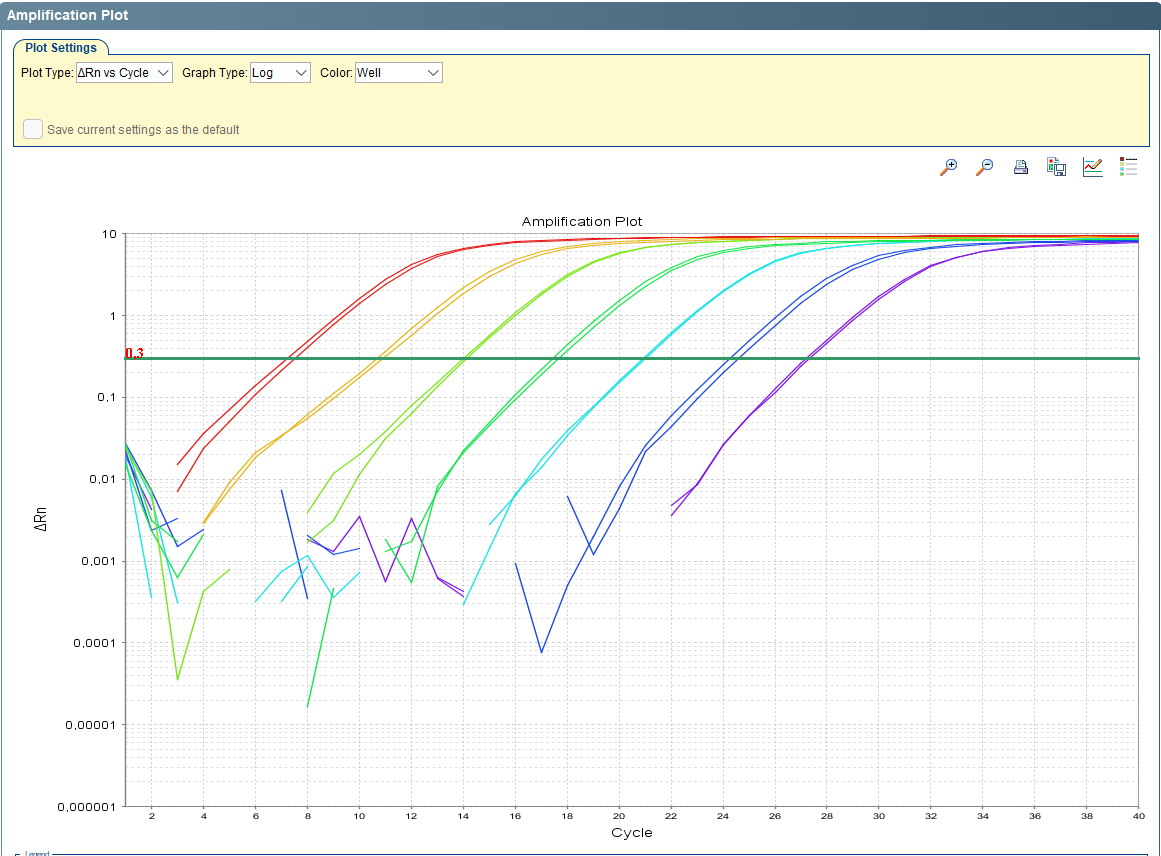


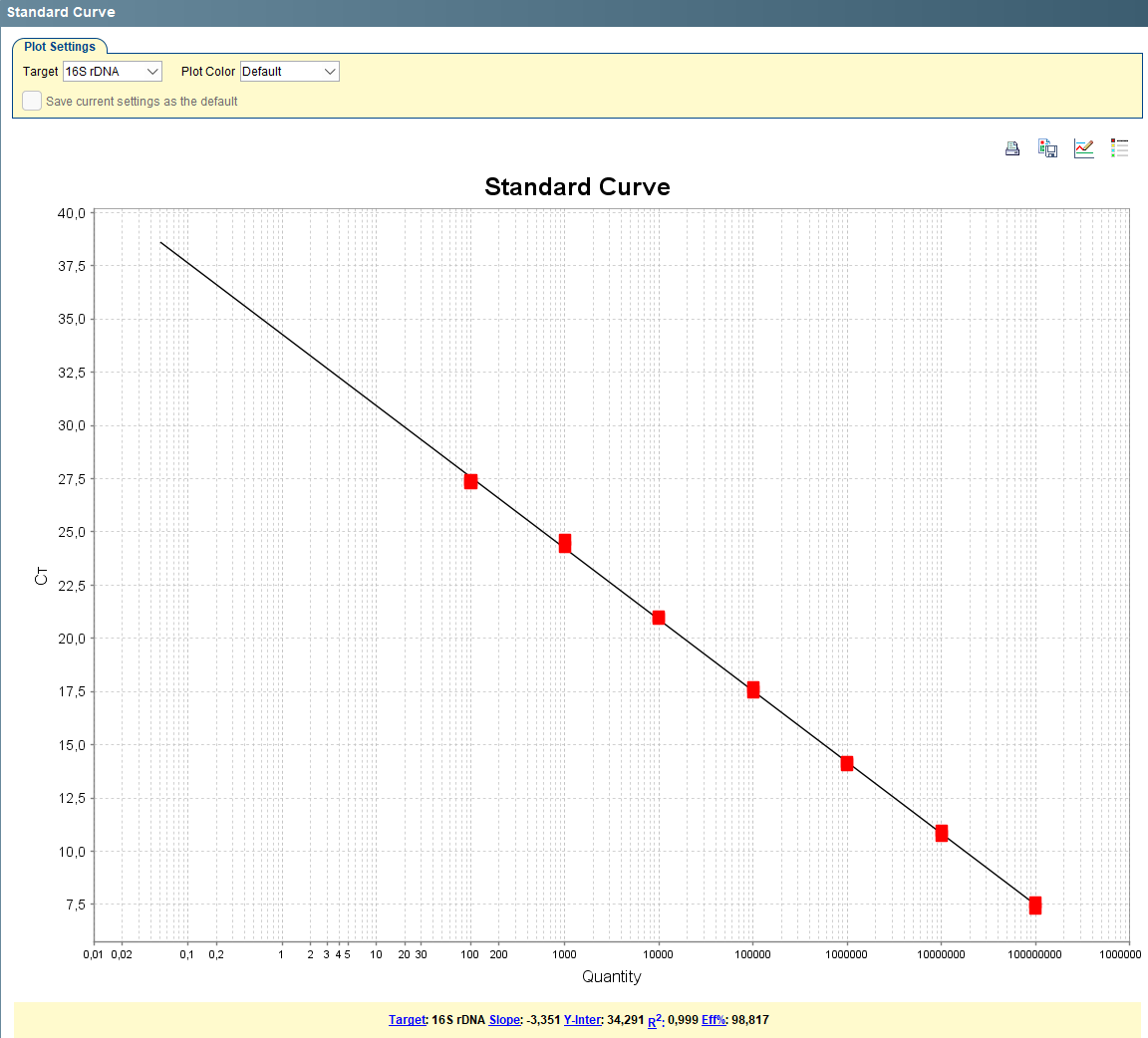


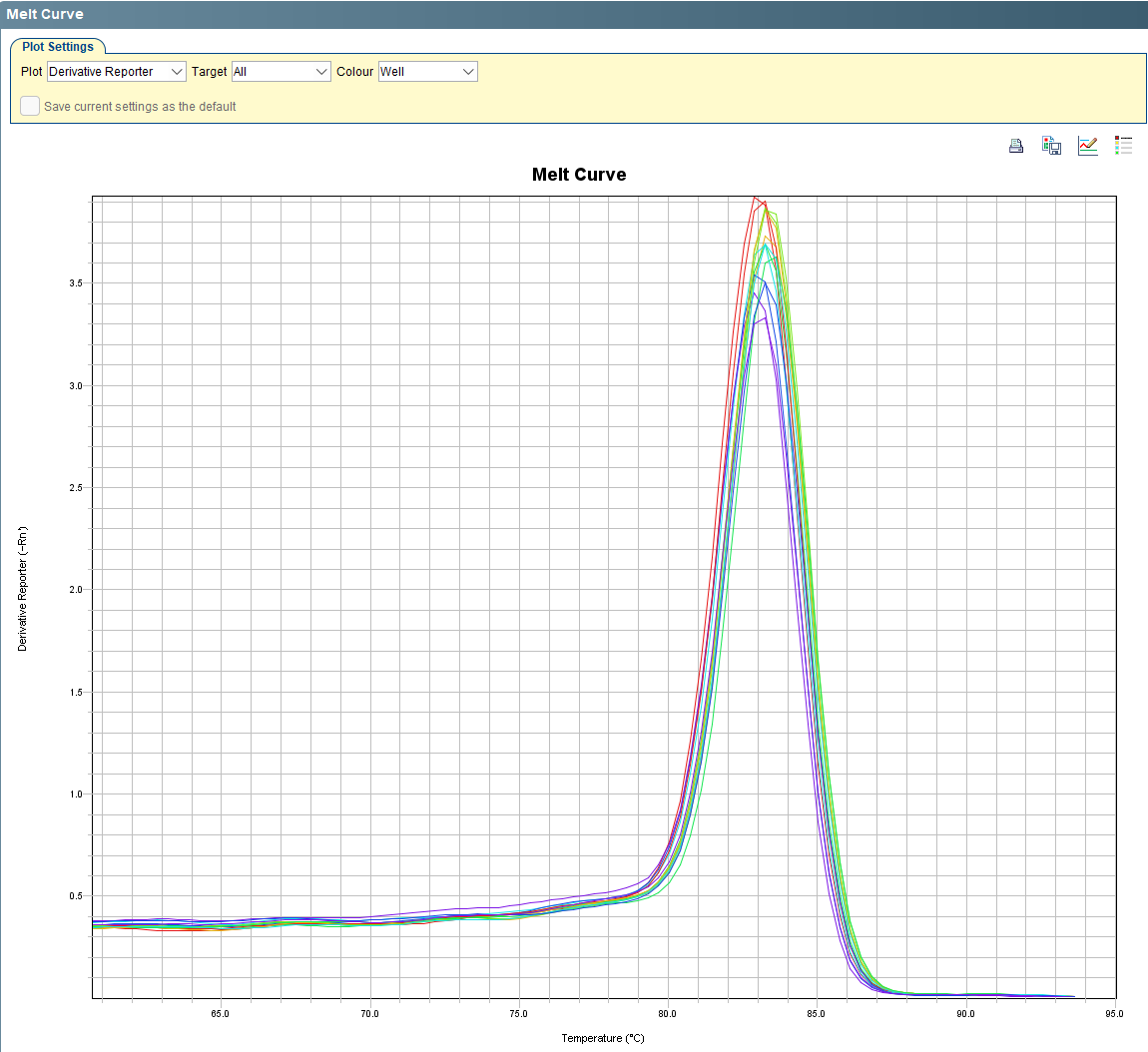


1. ***Escherichia coli***

**
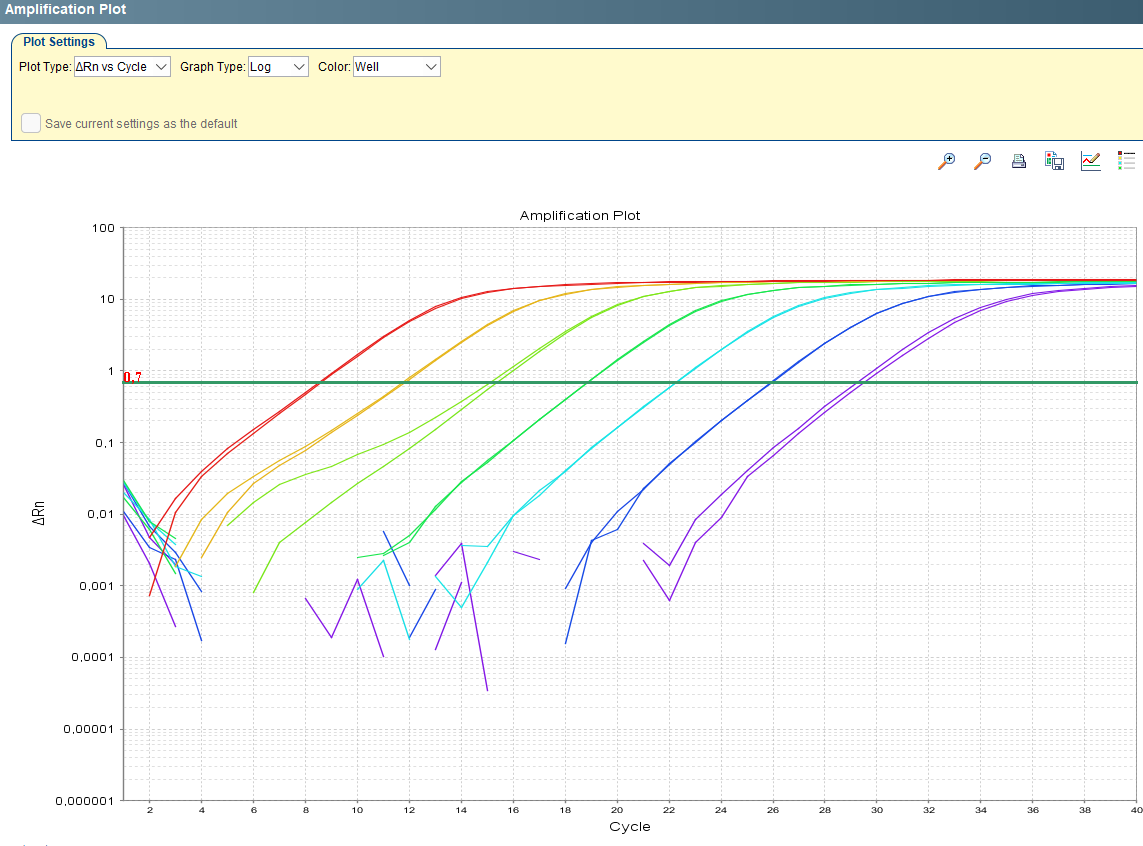
**

**
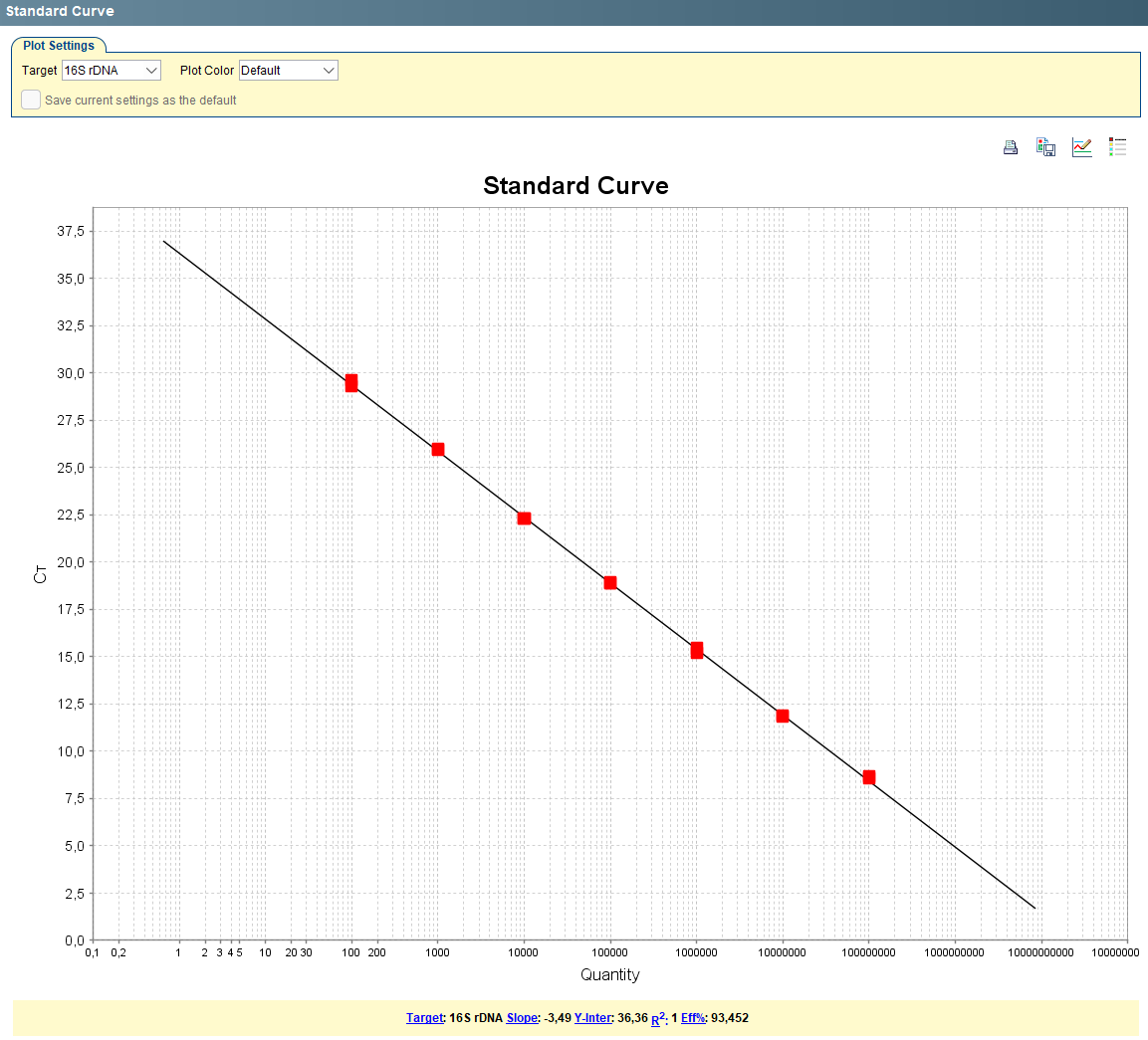
**

**
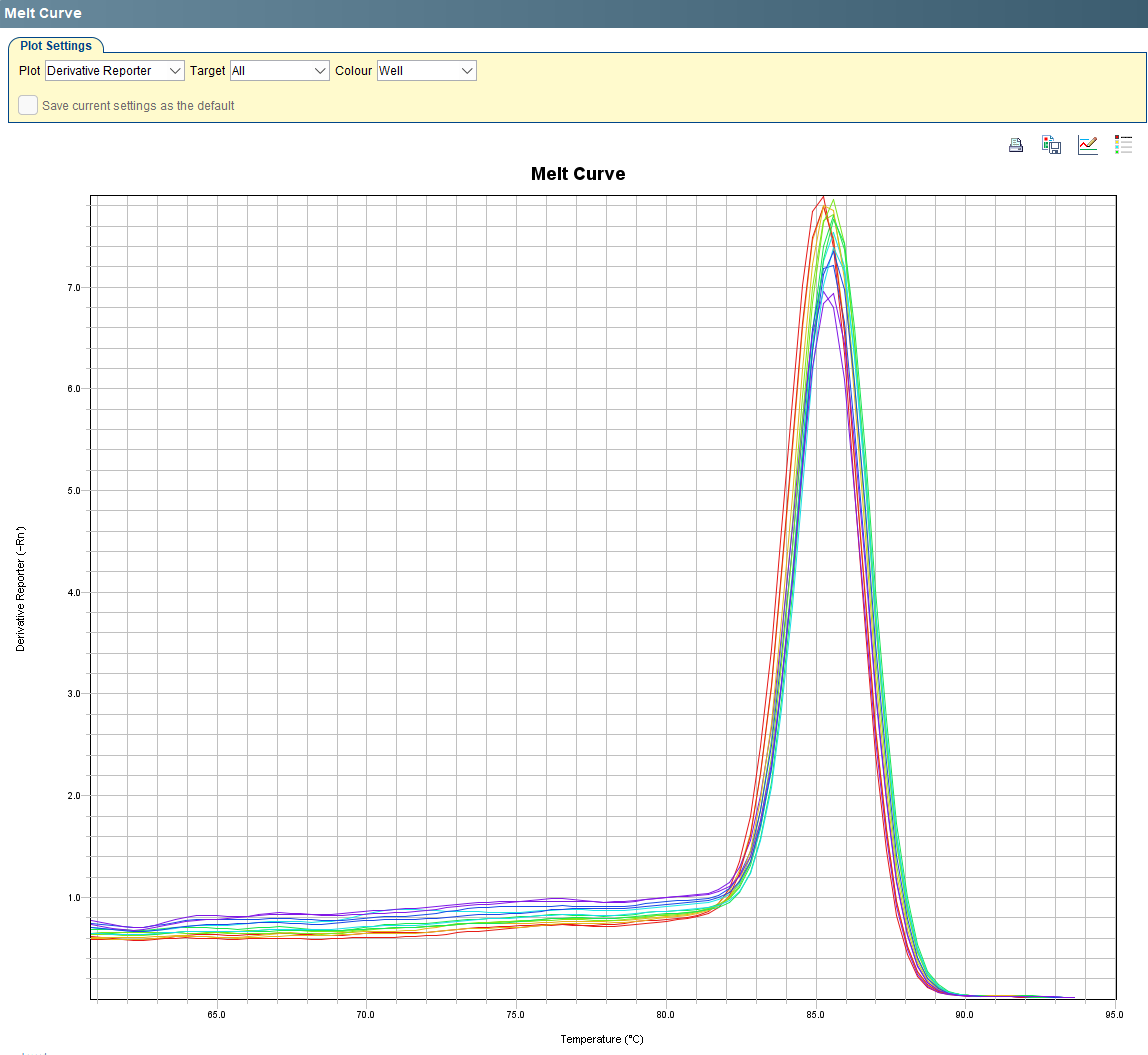
**
